# The interaction of Synapsin 2a and Synaptogyrin-3 regulates fear extinction in mice

**DOI:** 10.1172/JCI172802

**Published:** 2024-01-04

**Authors:** Xi-Ya Shen, Juan Zhang, He-Zhou Huang, Shao-Dan Li, Ling Zhou, Shi-Ping Wu, Cheng Tang, Xian Huang, Zhi-Qiang Liu, Zi-Yuan Guo, Xiang Li, Heng-Ye Man, You-Ming Lu, Ling-Qiang Zhu, Dan Liu

**Affiliations:** 1 Department of Pathophysiology, School of Basic Medicine, Tongji Medical College, Huazhong University of Science and Technology, Wuhan, Hubei, China.; 2 Center for Stem Cell and Organoid Medicine (CuSTOM), Division of Developmental Biology, Cincinnati Children’s Hospital Medical Center, Cincinnati, Ohio, USA.; 3 Department of Neurosurgery and; 4 Brain Research Center, Zhongnan Hospital of Wuhan University, Wuhan, China.; 5 Medical Research Institute, Wuhan University, Wuhan, Hubei, China.; 6 Department of Biology, Boston University, Boston, Massachusetts, USA.; 7 Department of Medical Genetics, School of Basic Medicine, Tongji Medical College, Huazhong University of Science and Technology, Wuhan, Hubei, China.

**Keywords:** Neuroscience, Neurological disorders

## Abstract

The mechanisms behind a lack of efficient fear extinction in some individuals are unclear. Here, by employing a principal components analysis–based approach, we differentiated the mice into extinction-resistant and susceptible groups. We determined that elevated synapsin 2a (Syn2a) in the infralimbic cortex (IL) to basolateral amygdala (BLA) circuit disrupted presynaptic orchestration, leading to an excitatory/inhibitory imbalance in the BLA region and causing extinction resistance. Overexpression or silencing of Syn2a levels in IL neurons replicated or alleviated behavioral, electrophysiological, and biochemical phenotypes in resistant mice. We further identified that the proline-rich domain H in the C-terminus of Syn2a was indispensable for the interaction with synaptogyrin-3 (Syngr3) and demonstrated that disrupting this interaction restored extinction impairments. Molecular docking revealed that ritonavir, an FDA-approved HIV drug, could disrupt Syn2a-Syngr3 binding and rescue fear extinction behavior in Syn2a-elevated mice. In summary, the aberrant elevation of Syn2a expression and its interaction with Syngr3 at the presynaptic site were crucial in fear extinction resistance, suggesting a potential therapeutic avenue for related disorders.

## Abstract 2

<p>Elevated Syn2a in the infralimbic cortex to the&nbsp;basolateral amygdala region&nbsp;disrupts presynaptic orchestration, leading to extinction resistance *via* interaction with Synaptogyrin-3.</p>

## Graphical abstract

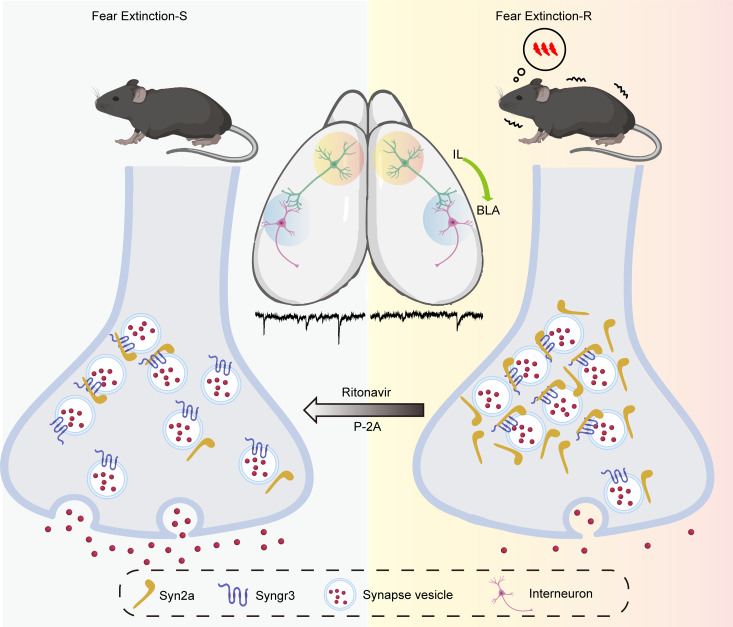

## Introduction

Posttraumatic stress disorder (PTSD) is a debilitating neuropsychiatric disorder (1) with complicated psychological and neurobiological mechanisms involving varied brain circuits mediating stress and fear responses (2–4), as well as diverse molecules related to synaptic function in key brain regions (5, 6). Since many individuals experience potentially traumatic events, even minor changes in their perception of life-threatening situations can significantly impact the prevalence of PTSD within the population (7). Previous studies have shown that approximately 20% of people who encounter such traumatic events develop PTSD, while others are able to overcome these experiences. This suggests that distinct populations may have varying levels of susceptibility to PTSD (8). Although the trigger event for PTSD is known, it is still unclear why some people develop PTSD after traumatic stimulation, but not others (9). Through many preclinical studies, researchers have identified that impaired fear extinction mechanisms after traumatic stress play an important role in the development and maintenance of PTSD (10, 11). Compared with healthy controls, individuals with PTSD display greater fear-potentiated startle (FPS) responses to the previously reinforced conditioned stimulus (CS) during the early and middle stages of extinction (1). Moreover, the impaired ability for fear inhibition was specifically found in individuals with PTSD in response to a CS that has not been reinforced by an unconditioned stimulus (US) (12). Some prospective studies even suggested that increased PTSD symptom severity is highly correlated with impaired fear extinction before traumatic stress (13). Therefore, understanding the mechanisms in fear extinction impairments will benefit the development of therapeutic strategies for PTSD.

Classical auditory fear conditioning is a form of associative learning that has been widely used in the study of fear memory and extinction in rodents (14). During fear conditioning, a neutral CS, such as an auditory cue, is associated with the US, such as an aversive shock (15). Extinction of fear occurs when the CS is repeatedly presented in the absence of the US, leading to diminished responses to cued conditioned fear (15). The lateral nucleus of the amygdala (LA) is a site showing experience-driven synaptic plasticity believed to be associated with CS-US (16). Evidence suggests that long-term potentiation (LTP) in sensory pathways from auditory CS projections to the LA underlies fear conditioning learning (17). The expression and extinction of fear memory involve synchronized activity in a network of highly conserved brain regions, including the basolateral amygdala (BLA), medial prefrontal cortex (mPFC), and ventral hippocampus (vHip). However, the underlying molecular mechanism is still unclear.

Previous evidence indicates that the prelimbic cortex (PL) in the mPFC mediates the expression of fear memory, while the infralimbic cortex (IL) participates in fear extinction by projecting to the amygdala (18–20). Studies have shown that fear extinction is associated with reduced synaptic efficacy in the projections from the mPFC to the principal neurons of the BLA, and the balance between excitation and inhibition in these projections shifts toward inhibition after fear extinction (3). Optogenetic stimulation of IL inputs into the amygdala during extinction training enhances extinction memory learning, while optogenetic or chemogenetic silencing of the IL impairs extinction learning (18, 20, 21). The inputs from the prefrontal cortex to the amygdala are regulated by inhibitory interneurons (22), and fear extinction has been found to increase the density of parvalbumin-positive interneurons (PV-IN) and cholecystokinin-positive interneurons (CCK-IN) around active fear neurons in the basal amygdala (23). In addition, fear conditioning and extinction can regulate inhibitory neurons to modulate freezing behavior (22). Deep brain stimulation (DBS) can modulate BLA activity and promote fear extinction in individuals with PTSD (24). High-frequency BLA stimulation also attenuates anxiety-like behaviors in PTSD model mice (25), highlighting the importance of BLA neuronal activity in fear extinction. However, the molecular processes underlying the projections from the IL to the BLA interneurons in fear extinction impairments are still unknown.

In this study, we established a principal components analysis–based (PCA-based) approach to divide mice into extinction-resistant and extinction-susceptible groups. We then demonstrated that presynaptic inhibition of the IL-BLA circuit disrupts the excitation/inhibition (E/I) balance in the BLA, causing resistance to fear extinction. Among a large number of presynaptic proteins, we found that elevated levels of Synapsin 2a (Syn2a), but not Synapsin 2b (Syn2b), play a critical role in mediating the presynaptic inhibition, disturbed E/I balance in the IL-BLA circuit, and impaired extinction. Using a series of biochemical screening approaches, we identified a conserved interaction between Syn2a and the 91–99 amino acid residues in Synaptogyrin-3 (Syngr3) via its specific H domain, which, in turn, blocks presynaptic vesicle release. Additionally, using an AI-assisted molecular docking approach, we determined that ritonavir, an FDA-approved drug for HIV infection, like blocking peptide P-2A, can effectively disrupt the binding of Syn2a with Syngr3 and enhance fear extinction in mice. Our study not only reveals the molecular mechanisms underlying fear extinction but also provides a mouse model and a potential drug candidate for PTSD therapeutics.

## Results

### Presynaptic inhibition of the IL-BLA circuit disrupts the E/I balance in the BLA of EXT-R mice.

**Figure 1:**
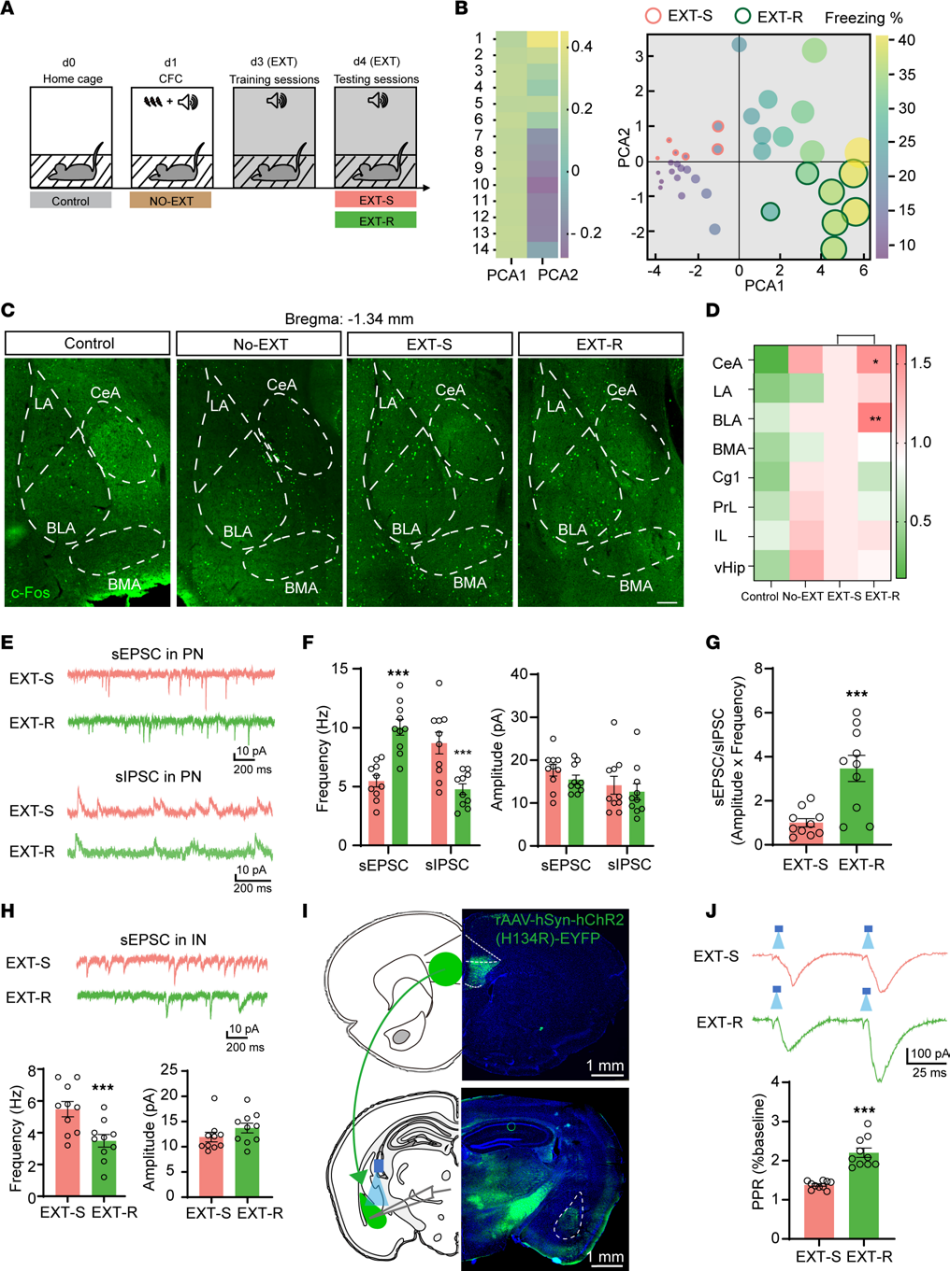
Presynaptic inhibition of the IL-BLA circuit disrupts the E/I balance in the BLA of EXT-R mice. (**A**) A schematic illustration of the fear memory extinction paradigm. (**B**) PCA plot of freezing times of 40 adult C57BL/6J mice during the extinction testing trials. (**C**) Representative confocal images of c-Fos staining in the amygdala for control, NO-EXT, EXT-S, and EXT-R mice. Scale bar: 100 μm. (**D**) The quantification of the number of c-Fos^+^ neurons in different brain regions of control, NO-EXT, EXT-S, and EXT-R mice (*n* = 3 mice per group). (**E**) Representative sEPSC and sIPSC traces recorded from EXT-S and EXT-R pyramidal neurons in the BLA. (**F**) Quantifications of frequencies and amplitudes of sEPSC and sIPSC. (**G**) Quantification of E/I ratios (sEPSC frequency × amplitude/ sIPSC frequency × amplitude) (*n* = 10 neurons from 3 mice per group). (**H**) Representative sEPSC traces recorded in BLA interneurons of EXT-S and EXT-R mice and the quantitative analysis (*n* = 10 neurons from 3 mice per group). (**I**) Representative photographs of ChR2-EYFP fluorescence at the viral IL injection site (upper) and ChR2-EYFP expressing afferent IL axon in the BLA (lower). (**J**) Paired-pulse ratios obtained from blue light–evoked excitatory postsynaptic current (EPSC) amplitudes reveal increased facilitation in EXT-R when compared with EXT-S mice. *n* = 10 cells (from 3 mice) per group. Statistical analyses among multiple groups were conducted using 1-way (**D**) or 2-way ANOVA (**F**) followed by Bonferroni post hoc tests, whereas an unpaired 2-tailed *t* test was conducted for comparing 2 groups (**G**, **H**, and **J**). *P < 0.05, **P < 0.01, and ***P < 0.001. Values are presented as mean ± SEM.

**Figure 2:**
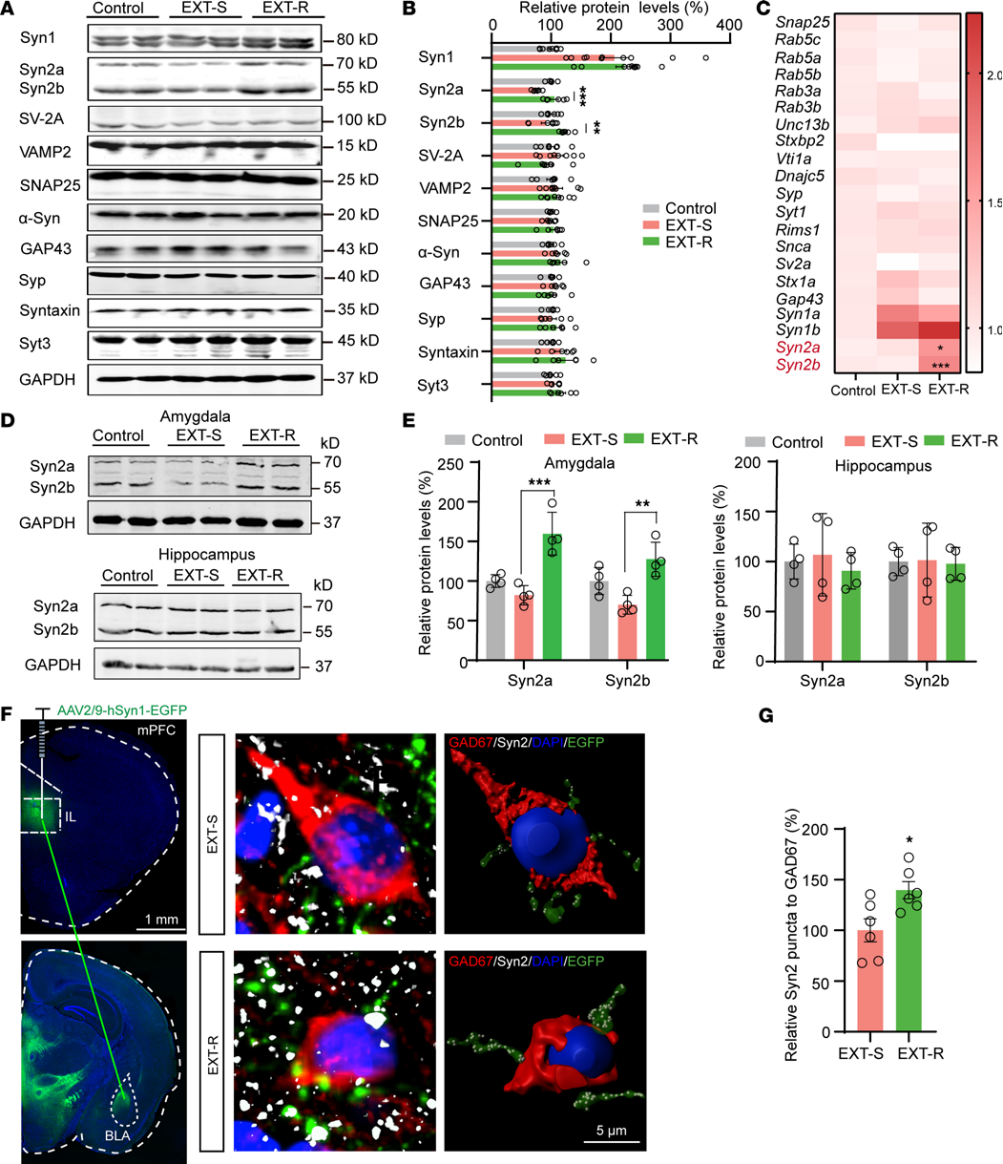
Upregulation of Syn2 in the IL is correlated with the presynaptic suppression of the IL-BLA circuit. (**A** and **B**) Representative Western blots (**A**) and quantification (**B**) for the protein levels of 11 presynaptic proteins in control, EXT-S, and EXT-R mice (*n* = 4 independent experiments). (**C**) qPCR analysis for the mRNA levels of 21 presynaptic proteins in control, EXT-S, and EXT-R mice (*n* = 3 per group, normalized to control). (**D** and **E**) Western blot analysis (**D**) and quantification (**E**) of Syn2a and Syn2b level expression in the presynaptic fraction of amygdala and hippocampus in control, EXT-S, and EXT-R mice (*n* = 3 independent experiments). (**F**) The mice were injected with AAV2/9-hSyn1-EGFP in the IL and then BLA slices from EXT-S and EXT-R mice were prepared to stain with anti-GAD67 (red) and anti-Syn2 (white). The nucleus was visualized by DAPI (blue). Representative images of virus infection (left), triple immunofluorescence (middle), and 3D reconstruction (right) were shown. Scale bars: 1 mm (left) and 5 μm (middle and right). (**G**) Relative average intensity of Syn2 puncta that overlap with green signals from IL to GAD67^+^ (upper) cells (*n* = 6, normalized to control). Statistical analyses among multiple groups were conducted using 1-way ANOVA followed by Bonferroni post hoc tests (**B**, **C**, and **E**), whereas an unpaired 2-tailed *t* test was conducted for comparing 2 groups (**G**). *P < 0.05, **P < 0.01, and ***P < 0.001. Values are presented as mean ± SEM.

To replicate the impaired fear extinction observed in individuals with PTSD, we conducted the fear extinction paradigm as previously reported (26) (Figure 1A) (see fear conditioning/extinction paradigm in Methods) by using a cohort of 40 mice. We then performed a PCA based on the freezing times recorded in 14 trials of testing sessions. We observed that approximately 17% of the mice were in a distinct group that was far from the original point and exhibited longer freezing times in the test trials, indicating impairments in fear extinction. We therefore named these mice as extinction resistant (EXT-R) mice. Conversely, we selected the 15% of mice with the lowest freezing times and defined them as extinction susceptible (EXT-S) mice (Figure 1B and Supplemental Figure 1, A and B; supplemental material available online with this article; https://doi.org/10.1172/JCI172802DS1). Given that several brain regions have been implicated in fear extinction, including the amygdala (especially the BLA and central amygdala [CeA]), the vHip, and the mPFC, we compared neuronal activity in these areas in the control, no extinction (NO-EXT), EXT-S, and EXT-R mice using c-Fos staining (Figure 1C and Supplemental Figure 1C). We observed that fear memory required the activation of many brain regions as reported previously (27), and there were more c-Fos positive neurons in the CeA and BLA regions in the EXT-R group than in the EXT-S group (Figure 1D), suggesting abnormal neuronal activation in the amygdala of EXT-R mice. It is well-established that the E/I balance in the BLA region plays a vital role in neuron activation during extinction (3). We aimed to determine whether the aberrant neuronal activation in the BLA was due to altered E/I balance. Therefore, we costained the brain slices with antibodies against c-Fos and 2 markers, Glutamate Decarboxylase 67 (GAD67) and CaMKII, which indicate inhibitory and excitatory neurons, respectively. We found that the number of GAD67^+^/c-Fos^+^ positive neurons was reduced, whereas the number of CaMKII^+^/c-Fos^+^ positive neurons was increased in the BLA of EXT-R mice (Supplemental Figure 2, A and B). Moreover, the frequency of spontaneous excitatory postsynaptic currents (sEPSCs) was increased, while the frequency of spontaneous inhibitory postsynaptic current (sIPSC) was reduced in BLA pyramidal neurons of EXT-R mice compared with those of the EXT-S mice. However, no difference was found in the amplitude of sEPSCs and sIPSCs between the 2 groups (Figure 1, E and F). Additionally, the E/I ratio in BLA pyramidal neurons was elevated (Figure 1G), while the sEPSC frequency in interneurons of BLA was reduced (Figure 1H) in EXT-R mice. These results indicate that excitatory pyramidal neurons were activated, while the inhibitory neurons were suppressed in the BLA of EXT-R mice. We then tested whether the inhibitory circuit from the IL to BLA interneurons, which is implicated in the fear extinction (3), was deregulated in EXT-R mice. To this end, we injected AAV particles of hSyn-ChR2-EYFP fusion protein into the IL region of EXT-S and EXT-R mice. After 4 weeks, the EYFP fluorescence was observed in the soma of neurons in the IL regions and the axon terminals that project to the BLA (Figure 1I). By activating ChR2-expressing axons with local blue light stimulation in the BLA, we recorded paired-pulse ratio (PPR) by whole-cell recordings and found an increased PPR ratio in the EXT-R mice (Figure 1J). Based on these findings, we proposed that disruption of presynaptic orchestration in the IL-BLA circuit plays a crucial role in the impairment of fear memory extinction in the EXT-R mice. Therefore, we aimed to understand the underlying molecular mechanisms that mediate the presynaptic inhibition of IL-BLA circuit. To achieve this, we examined the 21 well known presynaptic proteins (28) in the mPFC and found that both the mRNA and protein levels of Syn2a and Syn2b were substantially increased in the EXT-R mice (Figure 2, A–C and Supplemental Figure 3, A and B). Immunofluorescence revealed that there were more Syn2^+^ puncta around the IL neurons of the EXT-R mice (Supplemental Figure 3C). Meanwhile, we found that in EXT-R mice, Syn2a/b proteins were increased in the presynaptic fraction of the amygdala but not the hippocampus, though both received projections from the IL (Figure 2, D and E). Moreover, the 3D reconstruction of immunofluorescence data suggested that Syn2 puncta were dramatically increased in the axon terminals originating from the IL onto the GAD67^+^ neurons but not CaMKII^+^ neurons in the amygdala of EXT-R mice (Figure 2, F and G and Supplemental Figure 3, D and E). Considering the reduced neuronal activity in the GAD67^+^ neurons in the BLA, these data suggested that the upregulation of Syn2 may be related to the presynaptic suppression at the inhibitory neurons in the BLA of the IL-BLA circuit.

### Upregulation of Syn2a increases resistance to extinction via presynaptic inhibition of the IL-BLA circuit.

**Figure 3:**
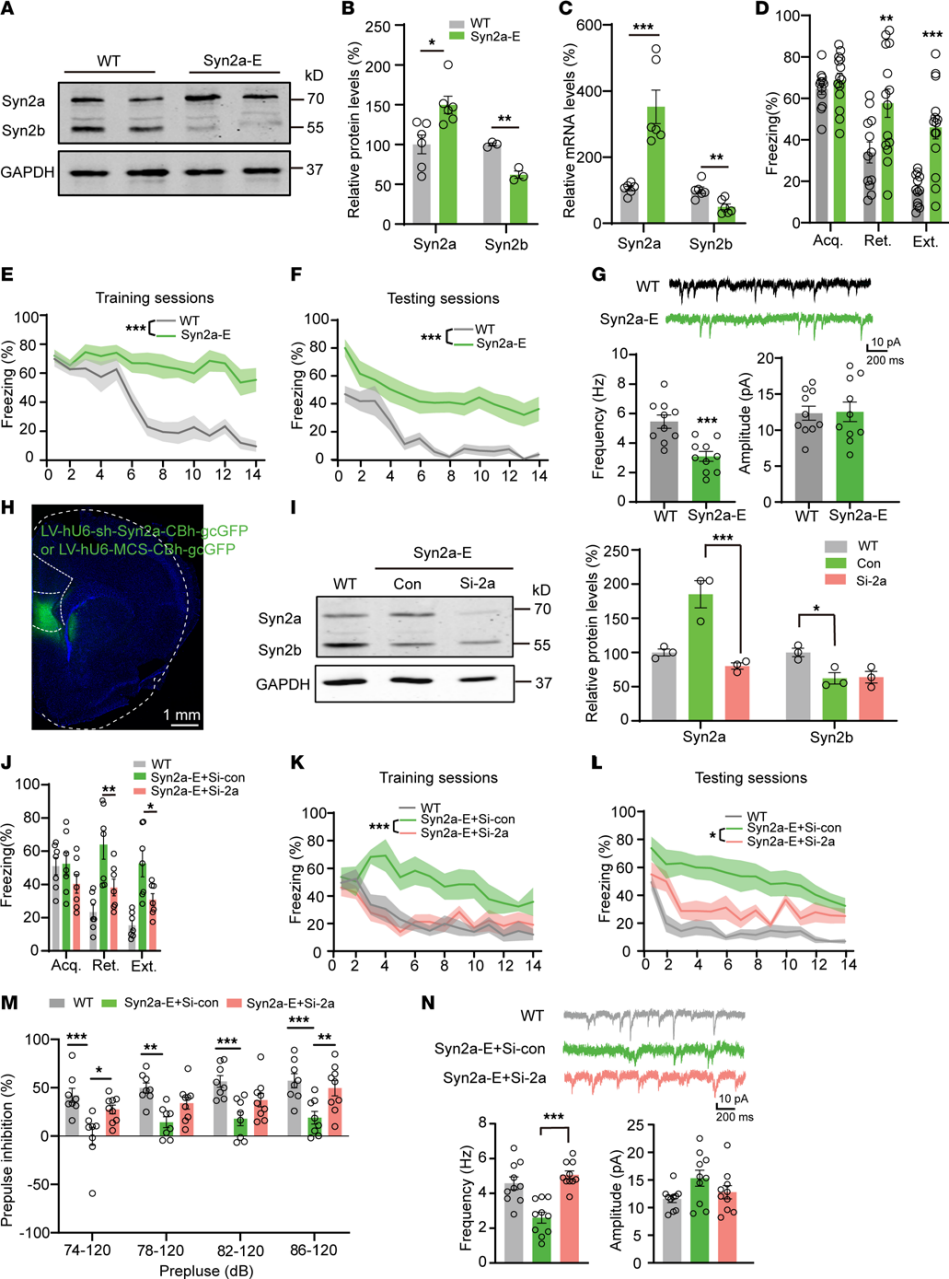
Syn2a is implicated in the extinction of fear memory. (**A** and **B**) Western blot analysis (**A**) and quantification (**B**) of Syn2a/b protein expression levels in the mPFC of WT and Syn2a-E mice (*n* = 3 mice). (**C**) Quantitative PCR analysis of relative mRNA levels of *Syn2a/b* in the mPFC in WT and Syn2a-E mice (*n* = 3 mice). (**D**) Average freezing response for all trials during fear acquisition and fear extinction retrieval and extinction of WT and Syn2a-E mice. (**E** and **F**) Freezing response during the extinction training sessions (**E**) and testing sessions (**F**) of WT and Syn2a-E mice (*n* = 12–14 per group). (**G**) Representative traces and quantitative analysis of the frequency and amplitude of sEPSCs in BLA interneurons of WT and Syn2a-E mice (*n* = 10 neurons from 3 mice). (**H**) Representative photomicrographs of injection sites in the IL. Scale bar: 1 mm. (**I**) Representative blots (left) and quantification (right) of Syn2a/b proteins from mPFC homogenates in WT, and control or si-Syn2a virus infected Syn2a-E mice (*n* = 3 per group). (**J**) Average freezing response for all trials during fear acquisition, fear extinction retrieval, and extinction**.** (**K** and **L**) Freezing response during the extinction training sessions (**K**) and testing sessions (**L**) (*n* = 7 per group). (**M**) Comparisons of percentages of PPI of startle responses with different startle amplitudes (*n* = 8–9). (**N**) Representative traces and average data of the frequency and amplitude of sEPSCs from BLA interneurons (*n* = 10 neurons from 3 mice). Statistical analyses among multiple groups were conducted using 1-way (**I** and **N**) or 2-way ANOVA (**B**, **C**, **D**, **J**, and **M**) followed by Bonferroni post hoc tests, whereas an unpaired 2-tailed *t* test was conducted for comparing 2 groups (**E**–**G**, **K**, and **L**). *P < 0.05, **P < 0.01, and ***P < 0.001. Values are presented as mean ± SEM.

**Figure 4:**
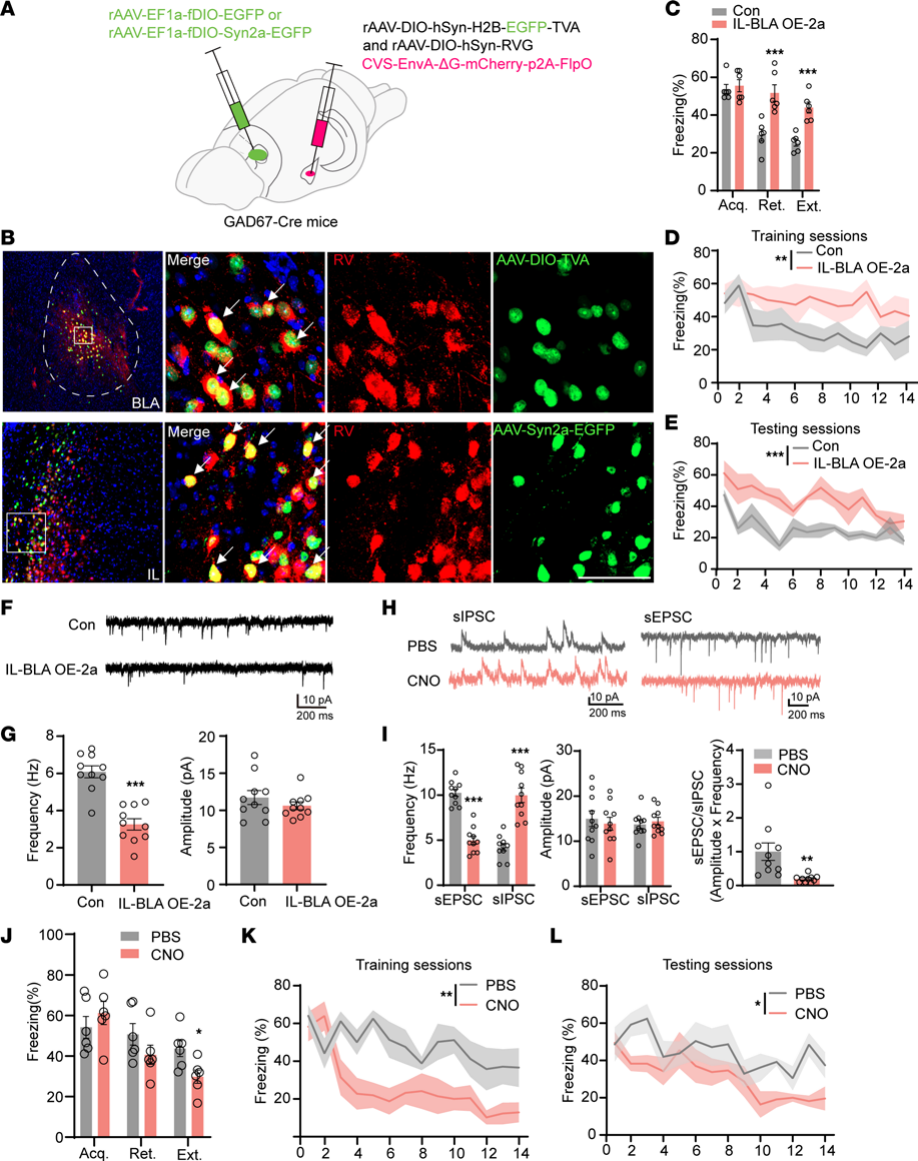
IL-BLA circuit–specific Syn2a overexpression induces E/I imbalance in the BLA of EXT-R mice. (**A**) A schematic illustration of the virus-induced dual-cre-loxp system to overexpress Syn2a specifically in the IL-BLA circuit. (**B**) Top, starter cells (yellow) in the BLA (colabeling of red [RV] and green [helper viruses]). Bottom, RV-labeled neurons in IL colocalized with the overexpressed Syn2a (green). Scale bar: 100 μm. (**C**) Average freezing response for all trials during fear acquisition, extinction retrieval, and extinction of IL-BLA OE-2a and control mice. (**D** and **E**) Freezing response during the extinction training sessions (**D**) and testing sessions (**E**) of IL-BLA OE-2a and control mice (*n* = 6 per group). (**F**) Representative traces of sEPSCs from BLA interneurons infected with control or IL-BLA OE-2a virus at IL. (**G**) Average data show that the frequency and amplitude of sEPSCs of BLA interneurons in IL-BLA OE-2a mice compared with control mice (*n* = 10 neurons from 3 mice per group). (**H**) Representative sEPSC and sIPSC traces recorded from PBS-injected and CNO-injected mice pyramidal neurons in BLA (*n* = 10 neurons from 3 mice per group). (**I**) Quantifications of frequencies and amplitudes of sEPSC and sIPSC and E/I ratios. (**J**) Average freezing response for all trials during fear acquisition and fear extinction retrieval and extinction of PBS- or CNO-injected mice (*n* = 6 per group). (**K** and **L**) Freezing response during the extinction training sessions (**K**) and testing sessions (**L**) of PBS- or CNO-injected mice (*n* = 6 per group). Statistical analyses among multiple groups were conducted using 2-way ANOVA followed by Bonferroni post hoc tests (**C**, **I**, and **J**), whereas an unpaired 2-tailed t test was conducted for comparing 2 groups (**D**, **E**, **G**, **K**, and **L**). *P < 0.05, **P < 0.01, and ***P < 0.001. Values are presented as mean ± SEM.

To examine the possible causal relationship between Syn2 elevation and fear extinction, we first employed a transgenic mouse model with elevated Syn2a but decreased Syn2b in the mPFC (Figure 3, A–C), as we reported previously (29), which we named as Syn2a–elevated (Syn2a-E) mice. We found that Syn2a-E mice had difficulty in fear memory extinction retrieval and extinction but not in acquisition (Figure 3, D–F). We also found that the frequency but not the amplitude of sEPSCs at BLA interneurons were substantially decreased in Syn2a-E mice (Figure 3G), suggesting that Syn2a-E mice fully replicated the extinction deficits and presynaptic dysfunction at BLA as seen in the EXT-R mice. To further confirm the critical role of Syn2a in the impaired fear extinction, we injected a lentivirus containing effective shRNA targeting mouse Syn2a (LV-hU6-shSyn2a-CBh-gcGFP) to knockdown Syn2a (Figure 3, H and I) in the IL neurons of Syn2a-E mice. We found that repression of Syn2a not only substantially decreased the freezing time (Figure 3, J–L), but also restored the reduced prepulse inhibition (PPI) (30) and the frequency of sEPSCs at BLA interneurons in Syn2a-E mice (Figure 3, M and N). Interestingly, knockdown Syn2a could affect the acquisition of fear memory and extinction retrieval in the WT mice (Supplemental Figure 4, A–C). Considering the Syn2b decrement was also apparent in the mPFC of Syn2a-E mice, we injected an AAV virus carrying the full-length mouse Syn2b (AAV2/8-hSyn1-Syn2b-mCherry, OE-2b) into the IL of Syn2a-E mice (Supplemental Figure 4D). However, no significant difference was detected in fear acquisition, extinction, and retrieval (Supplemental Figure 4, E and F). We also injected an AAV virus that packed the full-length mouse Syn2b (AAV2/8-hSyn1-Syn2b-mCherry, OE-2b) into the IL of WT mice and no significant difference was detected in fear acquisition, extinction, and retrieval (Supplemental Figure 4, G–I), which excludes the possible role of Syn2b decrement in the extinction deficits of Syn2a-E mice. To investigate whether the specific upregulation of Syn2a in the IL-BLA interneuron circuit affects fear memory extinction, we designed a dual-virus based Cre-loxp system to overexpress Syn2a in the IL neurons that project to BLA interneurons specifically (IL-BLA OE-2a) (Figure 4A). We first injected CVS-EnvA-ΔG-mCherry-p2A-Flpo plus rAAV-DIO-hSyn-H2B-EGFP-TVA and rAAV-DIO-hSyn-RVG virus into the BLA of GAD67-cre mice to express the Flpo recombinase in the IL neurons that specifically project to the BLA interneuron. After 4 weeks, we injected rAAV-EF1a-fDIO-EGFP or rAAV-EF1a-fDIO-Syn2a-EGFP virus into the IL to overexpress Syn2a in the IL neurons with Flpo expression (Figure 4B). Overexpression of Syn2a was confirmed by immunofluorescence staining (Supplemental Figure 5, A and B) and single cell quantitative PCR (Supplemental Figure 5C). We observed that mice with Syn2a overexpression (IL-BLA OE-2a) exhibited a marked increase in freezing time compared with the control group, without affecting the acquisition of fear memory (Figure 4, C–E). By whole cell patch clamp recordings, we found that the mean frequency, but not the amplitude, of sEPSCs at BLA interneurons was significantly decreased in IL-BLA OE-2a mice (Figure 4, F and G). Meanwhile, we also designed a recombinant AAV2/8 virus carrying the full-length mouse Syn2a cDNA tagged with mCherry and injected it into the IL of naive mice bilaterally (IL OE-2a) (Supplemental Figure 5, D and E). Four weeks after the virus injection, the mice were subjected to the cued fear memory extinction paradigm. We found the mice with Syn2a overexpression (IL OE-2a) displayed significantly increased freezing times compared with the control group without affect the acquisition of fear memory (Supplemental Figure 5, F and G). Additionally, chemogenetic activation of BLA inhibitory neurons rebuilt the E/I balance and rescued the extinction deficits in IL OE-2a mice to a great extent (Supplemental Figure 5H and Figure 4, H–L). To exclude the possible involvement of spatial and working memory in the effects of Syn2 overexpression, we employed the Morris water maze and novel object recognition tests. We found no deficit in Morris water maze acquisition or memory in control, Syn2a overexpressed (IL OE-2a), or Syn2b overexpressed (IL OE-2b) mice (Supplemental Figure 6, A–D). Further, we found no significant differences in control, IL OE-2a, or IL OE-2b mice in the novel object recognition test (Supplemental Figure 6E). To exclude the possible involvement of emotion abnormalities in the impaired fear extinction, we employed the open field test and elevated plus maze (EPM) tasks and found only Syn2b overexpression led to anxiety-like behaviors, as indicated by less time in the open arms in EPM (Supplemental Figure 6, F–H). To exclude the effect of the strong current on fear memory, we employed the fear conditioning/extinction paradigm of 0.5 mA foot shock. We found no deficit in fear memory acquisition in control, IL OE-2a mice of 0.5 mA foot shock (Supplemental Figure 6I). Together, these results suggested that elevation of Syn2a, not Syn2b in the IL led to the presynaptic inhibition and extinction resistance.

### Syn2a blocks the release of the presynaptic vesicle by binding with Syngr3 via its H domain.

**Figure 5:**
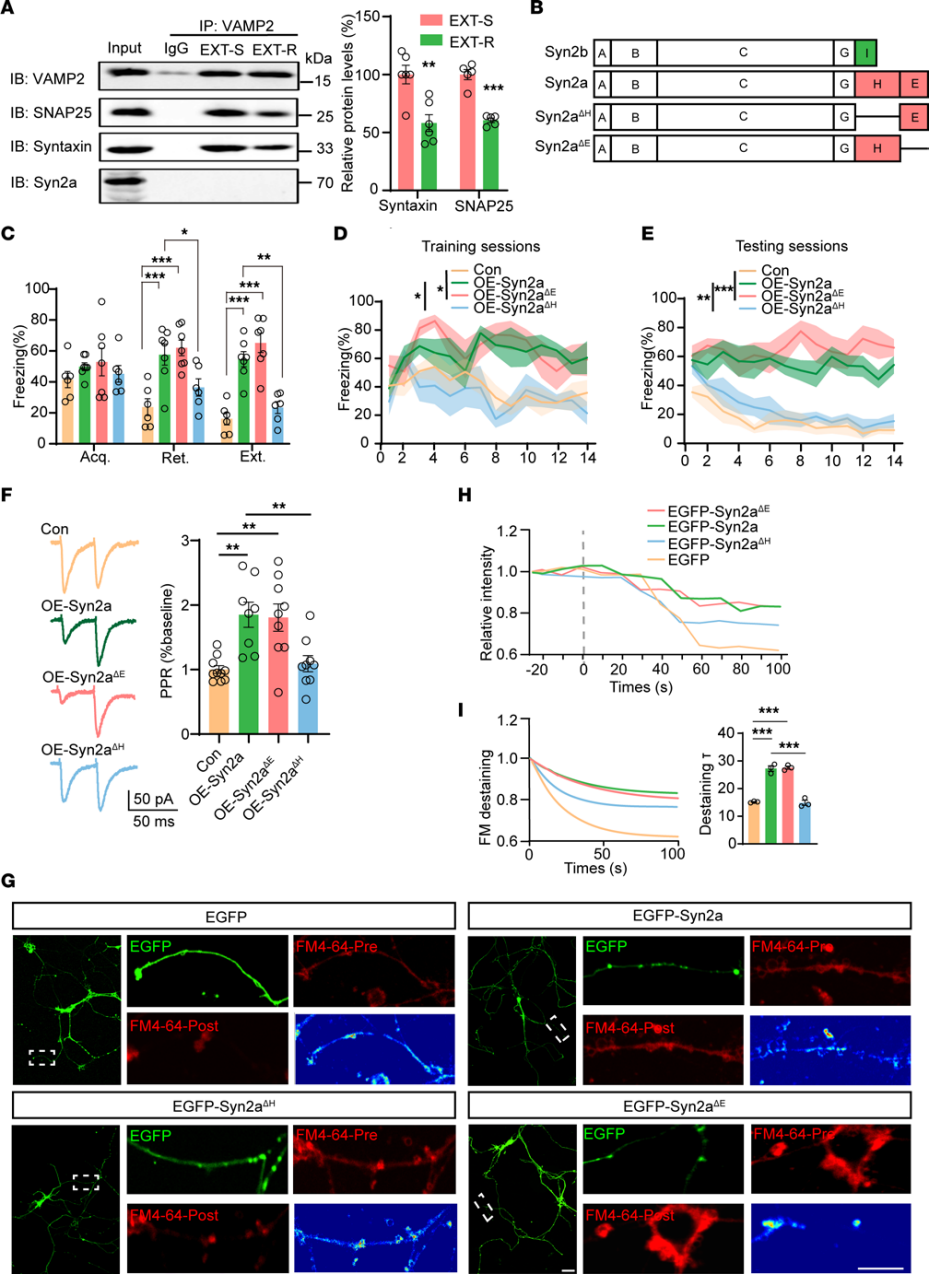
Syn2a blocked the presynaptic vesicle releasing via its H domain. (**A**) The presynaptic fraction of mPFC homogenates was prepared and then immunoprecipitated by using anti-VAMP2. The pellets were then subjected for immunoblot with the antibodies of anti-Syntaxin, anti-SNAP25 and anti-Syn2a. The representative blots (left) and the quantitative analysis (right) were shown (*n* = 3 per group). (**B**) Graphic diagram illustrating the differences in protein domains among WT Syn2b, Syn2a, and 2 Syn2a mutants. (**C**) Average freezing response for all trials during fear acquisition, extinction retrieval, and extinction of control, AAV-Syn2a, AAV-Syn2a-ΔH, and AAV-Syn2a-ΔE groups in mice. (**D** and **E**) Freezing response during the extinction training (**D**) and testing sessions (**E**) of control, AAV-Syn2a, AAV-Syn2a-ΔH, and AAV-Syn2a-ΔE groups in mice (*n* = 6–7 per group). (**F**) Paired-pulse ratios recorded from sEPSC amplitudes. (**G**–**I**) The primary cortical neurons were transfected with EGFP-C1, EGFP-Syn2a, EGFP-Syn2a-ΔH, or EGFP-Syn2a-ΔE plasmid at DIV 7. Then the FM4-64 releasing experiment was performed with a time-series based procedure at DIV 14. The representative confocal images (**G**) were shown. Pre: before the 90 mM KCl stimulation; Post: after the 90 mM KCl stimulation; pseudo color images indicate the change in FM4-64 fluorescent values (post–pre). Scale bar: 100 μm. The kinetics of FM4-64 fluorescence recorded from approximately 20 seconds before to approximately100 seconds after K+ stimulation are shown in **H**. The single-exponential decay functions were fitted to the diagrams with fluorescence intensity changes of FM4-64 analyzed by Image J software (left) and the time constant τ (right) were analyzed (**I**) (*n* = 3 independent experiments). Statistical analyses among multiple groups were conducted using 1-way (**D**, **E**, **F**, and **I**) or 2-way (**A** and **C**) ANOVA followed by Bonferroni post hoc tests. *P < 0.05, **P < 0.01, and ***P < 0.001. Values are presented as mean ± SEM.

**Figure 6:**
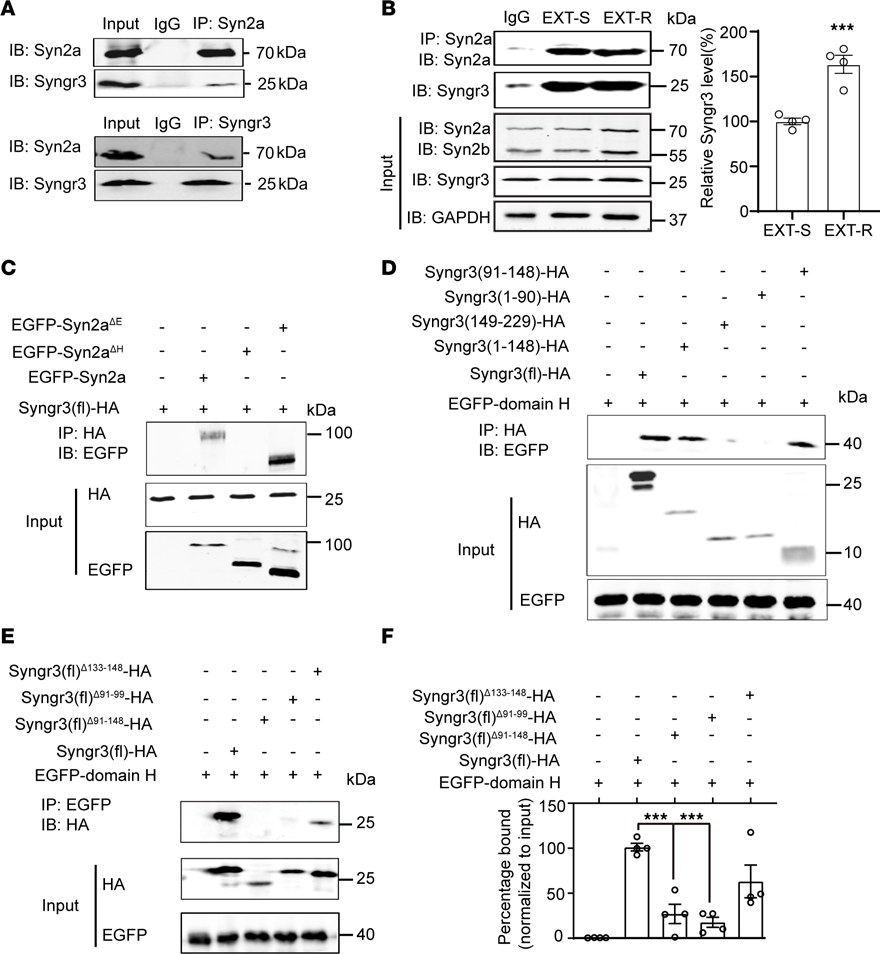
Syn2a interacts with AA91-99 of Syngr3 via domain H. (**A**) Co-IP of Syn2a and Syngr3 from the presynaptic fraction of amygdala homogenates of naive mice (*n* = 3 replicates). IP, immunoprecipitation; IB, immunoblotting. (**B**) Co-IP and quantitative analysis to evaluate the binding of Syn2a and Syngr3 from EXT-S and EXT-R mouse mPFC lysates (*n* = 3 replicates). (**C**) H293T cells were transiently transfected with the EGFP-Syn2a, EGFP-Syn2a-ΔH, EGFP-Syn2a-ΔE, and full-length Syngr3 [Syngr3(fl)-HA] plasmids. The cell lysates were collected and immunoprecipitated with an anti-HA antibody. Western blotting was performed using anti-HA and anti-EGFP antibodies. (**D**) H293T cell were transiently transfected with the Syngr3 (fl)-HA, Syngr3 (1-148)-HA, Syngr3 (149-229)-HA, Syngr3 (1-90)-HA or Syngr3 (91-148)-HA, and EGFP-domain H plasmids. The cell lysates were collected and immunoprecipitated with an anti-HA antibody. Western blot was performed by using anti-HA and anti-EGFP antibodies. (**E**) H293T cells were transiently transfected with the Syngr3 (fl)-HA, Syngr3 (deletion 91-148)-HA, Syngr3 (deletion 91–99)-HA, Syngr3 (deletion 133–148)-HA, and EGFP-domain H plasmids. The cell lysates were collected and immunoprecipitated with an anti-EGFP antibody. Western blot was performed by using anti-HA and anti-EGFP antibodies. (**F**) Quantitative analysis of Co-IP using the anti-EGFP antibody (*n* = 3 replicates). Statistical analyses among multiple groups were conducted using 1-way ANOVA followed by Bonferroni post hoc tests (**F**), whereas an unpaired 2-tailed *t* test was conducted for comparing 2 groups (**B**). ***P < 0.001. Values are presented as mean ± SEM.

Although Syn2a is a well known presynaptic vesicle–related protein (31) and plays an important role in maintaining the vesicles in the reserve pool, little is known about how Syn2a could anchor the synaptic vesicles (SVs) away from the releasing site. Indeed, less Syntaxin and SNAP25, but not Syn2a, were found in the presynaptic fraction of amygdala that was immunoprecipitated by VAMP2 in EXT-R mice (Figure 5A and Supplemental Figure 7A), suggesting a reduced SNARE complex, the critical component for vesicle releasing in the presynaptic compartment (in which Syn2a is absent), in these animals. We then tried to explore the specific domain in Syn2a protein to preserve the vesicles. It is known that Syn2a shared domains A, B, C, and G with Syn2b but possesses 2 unique domains of H and E (32). We then constructed 2 mouse Syn2a plasmids lacking domain H (Syn2a-ΔH) or domain E (Syn2a-ΔE), separately (Figure 5B and Supplemental Figure 7, B–D). By overexpression of these mutants in the IL, we found that Syn2a-ΔE but not Syn2a-ΔH resulted in fear extinction deficits (Figure 5, C–E) and elevated PPR (Figure 5F). In the primary cultures, overexpression of Syn2a and Syn2a-ΔE, but not Syn2a-ΔH, significantly reduced the presynaptic vesicle releasing rate (Figure 5, G–I). These data strongly suggest that domain H is the key domain in Syn2a to exert the presynaptic inhibitory function. To identify how domain H of Syn2a anchors the synaptic vesicle, we performed the GST pull–down experiment followed by mass spectrometry analysis by using the GST-fused domain H of Syn2a (GST-Syn2a-DomH) (Supplemental Figure 8A). A total of 108 proteins were identified in which only Syngr3 and Ras-related protein Rab3a (Supplemental Figure 8, B and C) were proteins associated with the vesicle membrane (28). The coimmunoprecipitation (Co-IP) assays suggested that the full length of Syn2a indeed physically interacted with Syngr3 but not Rab3a in the presynaptic fraction of amygdala homogenates and in the mPFC homogenates (Figure 6A and Supplemental Figure 8, D and E). In the EXT-R mice, the binding affinity of Syn2a with Syngr3 was enhanced, but the level of Syngr3 was not changed (Figure 6B). Meanwhile, deletion of domain H but not domain E in Syn2a lost the capacity to bind with Syngr3 (Figure 6C and Supplemental Figure 8F). To understand the specific amino acid sequence in Syngr3 that binds with Syn2a, we then constructed a series of truncated mutants of mouse Syngr3 and coexpressed with domain H of Syn2a or the full length of Syn2a in H293T cells (Supplemental Figure 8G). We found that deletion of amino acids 91–99, but not other residues, substantially reduced the binding capacity of Syngr3 with domain H of Syn2a (Figure 6, D–F and Supplemental Figure 9, A–C), as well as the full length of Syn2a (Supplemental Figure 9, D–I). Importantly, the physical binding of Syn2a with Syngr3 and the key amino acids were conserved among mammals (Supplemental Figure 10). These data demonstrate that elevated Syn2a interacts with AA91–99 of Syngr3 via domain H.

### Blocking the Syn2a/Syngr3 interaction preserves presynaptic function and promotes fear extinction.

**Figure 7:**
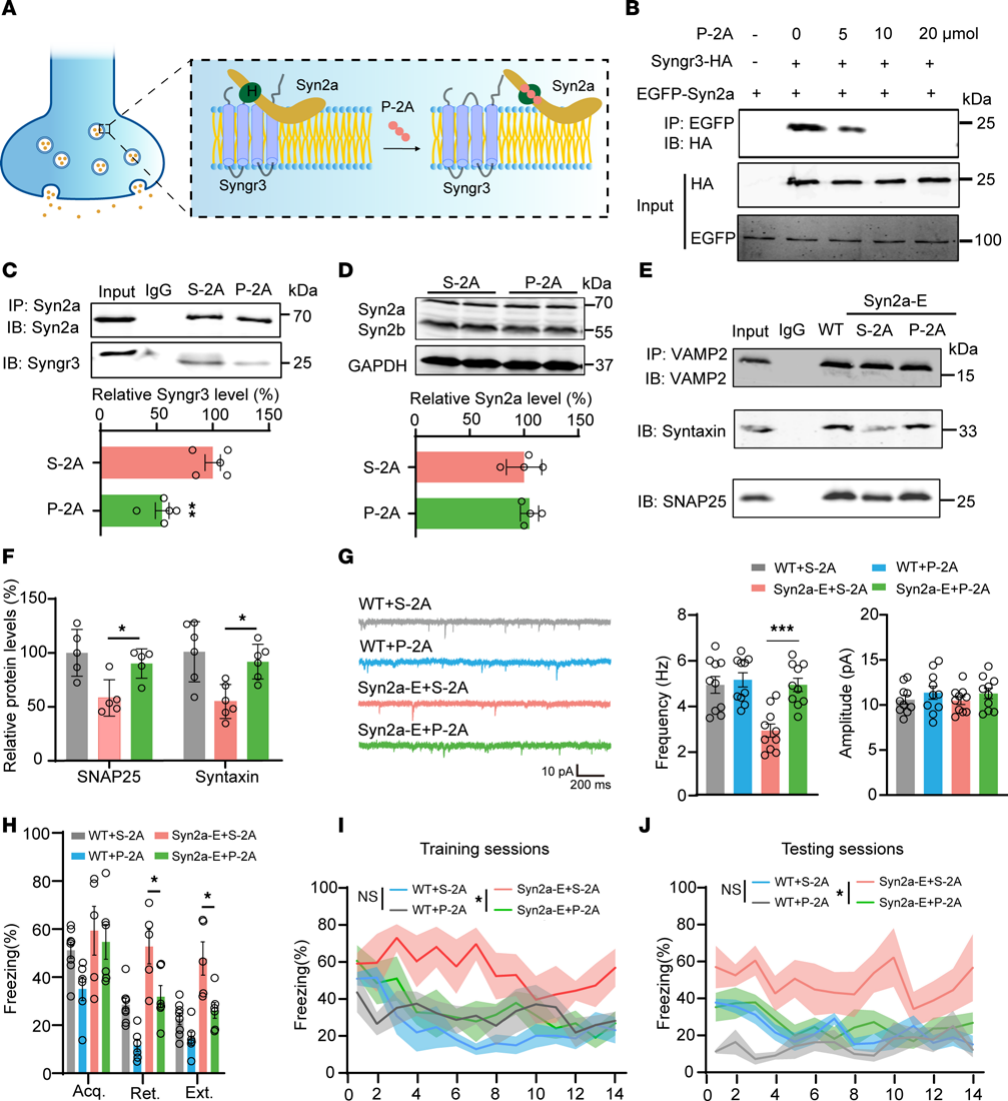
Blocking the Syn2a/Syngr3 interaction by P-2A preserves presynaptic function and promotes fear extinction. (**A**) A diagram illustrates that P-2A disrupts the binding of Syn2a with Syngr3 in the presynapse. (**B**) The H293T cells were transfected with EGFP-Syn2a and HA-Syngr3 plasmids. After 24 hours, cells were treated with P-2A for 12 hours. Cell lysates were then collected for Co-IP assay. (**C**) WT mice were intraperitoneally injected with P-2A or S-2A. mPFC homogenates were collected and immunoprecipitated (IP) by using the anti-Syn2a. Representative immunoblots (upper) and quantification (lower) (*n* = 5 per group). (**D**) Representative Western blots (upper) and quantification (lower) of the protein levels of Syn2a and Syn2b in P-2A and S-2A treated mice (*n* = 3 replicates). (**E**) The mPFC lysates were precipitated with rabbit VAMP2 antibody, and probed with anti-Syntaxin and anti-SNAP25. (**F**) Quantitative analysis of Co-IP (*n* = 5–6 per group). (**G**) Representative traces (left) and quantification (right) of sEPSCs from BLA interneurons in P-2A or S-2A treated WT or Syn2a-E mice (*n* = 10 neurons from 3 mice per group). (**H**) Average freezing response for all trials during fear acquisition, extinction retrieval, and extinction of WT + S-2A, WT + P-2A, Syn2a-E + S-2A, and Syn2a-E + P-2A groups in mice (*n* = 5–8 per group). (**I** and **J**) Freezing response during the extinction training sessions (**I**) and testing sessions (**J**) of WT + S-2A, WT + P-2A, Syn2a-E + S-2A, and Syn2a-E + P-2A groups in mice (*n* = 5–8 per group). Statistical analyses among multiple groups were conducted using 1-way (**F** and **G**) or 2-way (**H**) ANOVA followed by Bonferroni post hoc tests, whereas an unpaired 2-tailed *t* test was conducted for comparing 2 groups (**C**, **D**, **I**, and **J**). *P< 0.05, **P < 0.01, ***P < 0.001. Values are presented as mean ± SEM.

**Figure 8:**
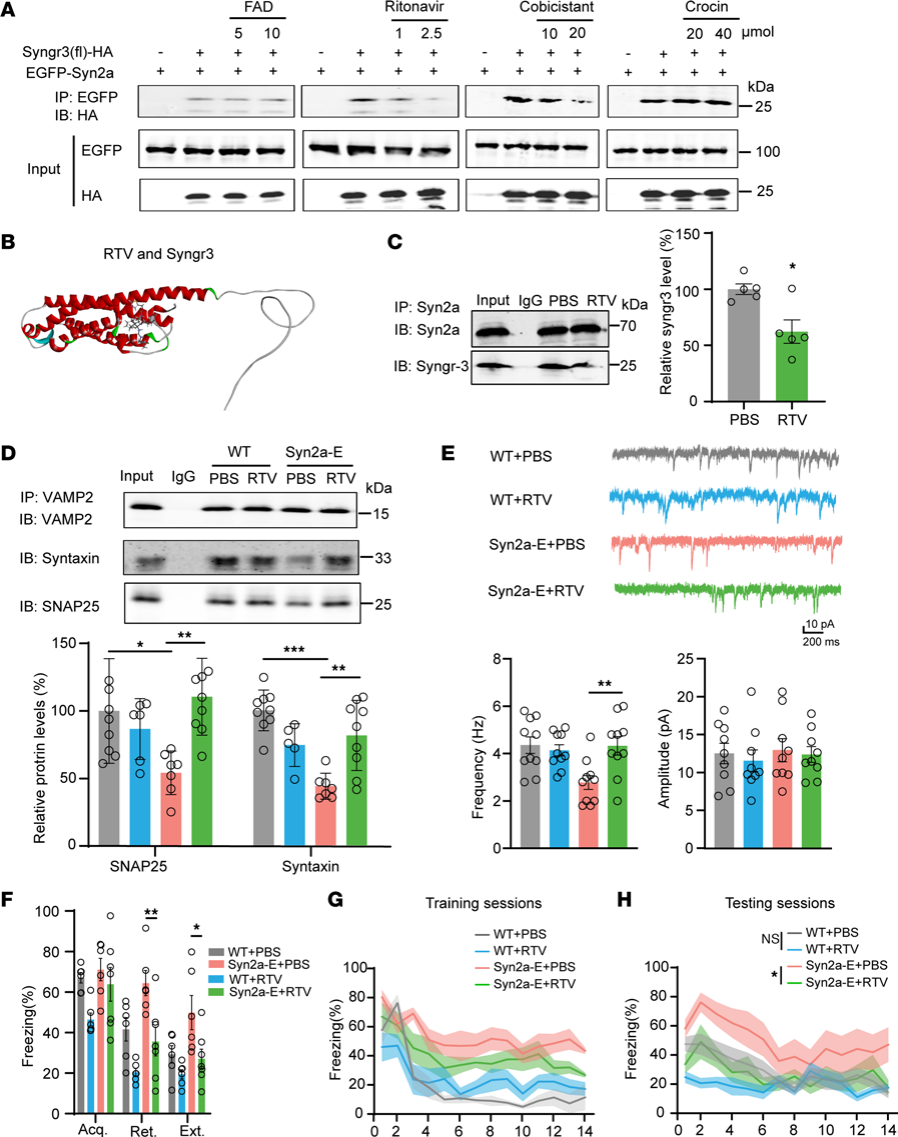
Ritonavir blocks the Syn2a/Syngr3 interaction to preserve presynaptic function and promote fear extinction. (**A**) The H293T cells were transfected with EGFP-Syn2a and HA-Syngr3 plasmids. After 24 hours, the cells were treated with FAD, ritonavir, cobicistat, or crocin with the indicated concentration for 12 hours. Then, the cell lysates were collected for co-IP assay. (**B**) A diagram of molecular docking of ritonavir on Syngr3. Syngr3 is labeled in red while the ritonavir is labeled in gray. (**C**) WT mice were intraperitoneally injected with ritonavir or PBS at the dose of 5 mg/kg 4 times. The mPFC homogenates were collected and immunoprecipitated (IP) by using the anti-Syn2a. Then, the pellets were used for Western blot (IB) analysis by using the antibodies indicated. Representative immunoblots (upper) and quantification (lower) (*n* = 4 per group). (**D**) mPFC lysates were precipitated with rabbit VAMP2 antibody, and probed with anti-Syntaxin anti-SNAP25. Representative immunoblots and quantification (*n* = 4 per group). (**E**) Representative traces and quantification of the frequency and amplitude of sEPSCs from BLA interneurons in ritonavir or PBS treated WT or Syn2a-E mice (*n* = 10 neurons from 3 mice per group). (**F**) Average freezing response for all trials during fear acquisition, extinction retrieval, and extinction of WT + PBS, WT + ritonavir, Syn2a-E + PBS, and Syn2a-E + ritonavir groups in mice (*n* = 6–7 per group). (**G** and **H**) Freezing response during the extinction training sessions (**G**) and testing sessions (**H**) of WT + PBS, WT + ritonavir, Syn2a-E + PBS, and Syn2a-E +r itonavir groups in mice (*n* = 6–7 per group). Statistical analyses among multiple groups were conducted using 1-way (**D** and **E**) or 2-way (**F**) ANOVA followed by Bonferroni post hoc tests, whereas an unpaired 2-tailed *t* test was conducted for comparing 2 groups (**C**, **G**, and **H**). *P < 0.05, **P < 0.01, and ***P < 0.001. Values are presented as mean ± SEM.

We then asked whether blocking the interaction between Syn2a and Syngr3 could rescue the presynaptic dysfunction and fear extinction resistance. To this end, we first designed a membrane-permeable peptide, P-2A, containing the Syngr3 91–99AA, and confirmed that 10 μM P-2A was able to disrupt the binding of Syn2a to Syngr3 in H293T cells (Figure 7, A and B). Intraperitoneal injection of P-2A was able to disrupt the binding of Syn2a to Syngr3 without affecting Syn2a/b protein levels in the mPFC (Figure 7, C and D and Supplemental Figure 11A). In addition, P-2A injection restored the reduced SNARE complex formation and preserved the frequency of sEPSCs in the BLA interneurons of Syn2a-E mice (Figure 7, E–G). Importantly, P-2A injection significantly rescued the impaired fear extinction in Syn2a-E mice but not WT mice (Figure 7, H–J). To seek potential compounds to disrupt the binding of Syn2a/Syngr3, we set the Syngr3 (91–99AA) as a binding pocket and performed a molecular docking strategy to identify potential compounds that are able to disrupt the binding of Syn2a/Syngr3 by using a structure-based virtual screening approach. About 20,000 compounds from a list of drugs approved in major jurisdictions (https://zinc.docking.org/) were analyzed to yield 938, 676 complexes and score each pose for physical complementarity to the Syn2a/Syngr3. After analyzing the top 100 poses, we selected 4 commercial compounds for further biological experiments (Supplemental Table 1). Indeed, these 4 compounds targeted the Syngr3 and formed hydrogen bonds with Syngr3 specifically (Supplemental Figure 11, B and C). However, only ritonavir and cobicistat were able to disrupt the binding of Syn2a/Syngr3 in vitro (Figure 8, A and B). Since cobicistat is an adjunctive drug, we focused on ritonavir. Intraperitoneal injection of ritonavir (5 mg/kg) was also able to disrupt the binding of Syn2a to Syngr3 without altering the Syn2a/b protein levels (Figure 8C and Supplemental Figure 11D). In addition, ritonavir injection restored the reduced SNARE complex formation (Figure 8D) and preserved the frequency of sEPSCs in the BLA interneurons of Syn2a-E mice (Figure 8E). Importantly, ritonavir injection significantly rescued the impaired fear extinction and the PPI ratio in Syn2a-E mice but didn’t affect the WT mice (Figure 8, F–H and Supplemental Figure 11E). We also administered ritonavir to the EXT-R mice and further confirmed that ritonavir injection significantly improved fear extinction (Supplemental Figure 12, A–F) and preserved sEPSC frequency in the BLA interneurons (Supplemental Figure 12G). Thus, disruption of Syn2a/Syngr3 interaction mitigated the presynaptic inhibition and impaired fear extinction in Syn2a-E mice.

## Discussion

**Figure 9:**
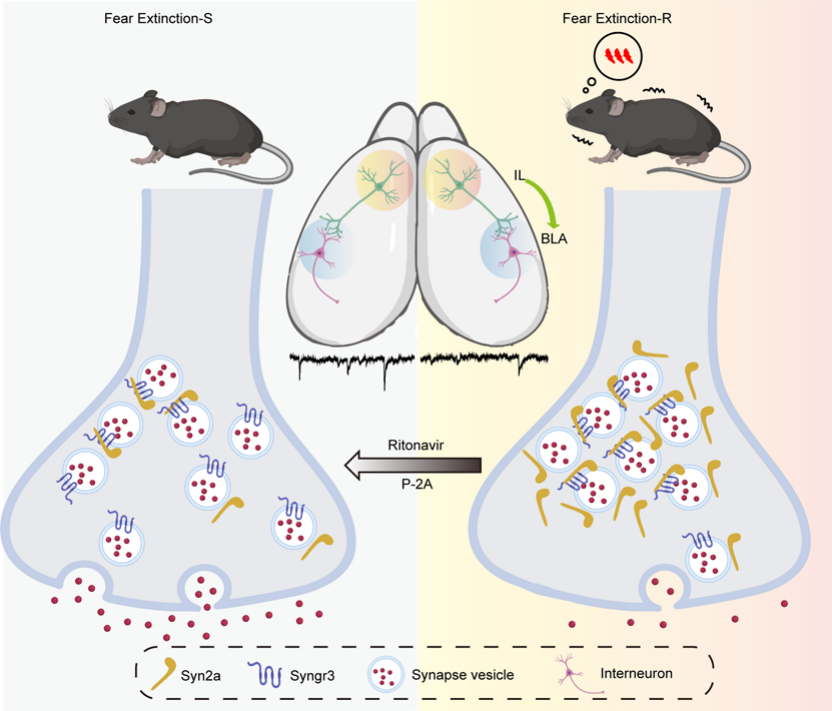
A working model illustrating how Syn2a remodels the IL-BLA circuit to slow fear extinction. Our findings demonstrate that the elevation of Syn2a interacts with the 91–99 amino acid residues in Syngr3 via its specific H domain, resulting in the blockade of presynaptic vesicle releasing. This blockade subsequently disrupts the E/I balance in the IL-BLA circuit and impairs extinction. Disrupting the binding affinity of Syn2a with Syngr3 using P-2A or Ritonavir, an FDA-approved drug for HIV infection, effectively improves fear extinction in mice.

In this study, we report that Syn2a interacts with the 91–99 amino acid residues in Syngr3 via its specific H domain. Elevated Syn2a blocks the presynaptic vesicle release and disrupts E/I balance in the IL-BLA circuit, leading to impairments in fear extinction. Blocking the interaction between Syn2a and Syngr3 by a BBB-permeable peptide, or Ritonavir, an FDA-approved drug for HIV infection, effectively preserves the E/I balance of BLA and improves fear extinction (Figure 9).

The communication between the mPFC and BLA has been implicated in normal fear extinction. For example, the firing rates of mPFC neurons were increased during memory extinction (33). Increased neural activity in the PL of the mPFC was observed in response to CS in rats and persistence of PL neuronal activation after extinction training correlates with extinction failure (34). Likewise, enhanced BLA LTP was associated with fear learning (35), while a decrease in BLA-evoked field potentials was found during fear extinction in rats (36). Further studies suggest that the inhibitory projection from mPFC to BLA plays an important role in fear extinction (37). Optogenetic stimulation of IL inputs into the amygdala in extinction training enhances extinction memory learning, while silencing of this projection impairs extinction learning (18, 20, 21). In addition, the prefrontal cortex seems to project both inhibitory neurons and excitatory neurons in the BLA. During normal fear extinction, excitatory synaptic efficacy from mPFC projections to BLA principal neurons (BLA/PN) is reduced while the projections to BLA GABAergic interneurons (BLA/IN) is unchanged, which leads to plasticity of inhibitory circuits in the BLA, a key mechanism in fear extinction (38). In line with these studies, we find that the inhibitory neurons were less activated while the excitatory neurons were over stimulated, suggesting an E/I imbalance in the BLA of EXT-R, but not the EXT-S mice. We also report that the efficiency of presynaptic inputs from mPFC to the inhibitory but not excitatory neurons of the BLA was dramatically reduced. Moreover, chemogenetic activation of GABAergic interneurons in the BLA or recovery of the presynaptic function restored the E/I balance and rescued the impaired fear extinction in Syn2a-E mice. These findings deepen our understanding of the role of BLA in fear extinction.

In our study, we also identified that abnormal upregulation of Syn2a, one of the important synapsin family members, in the IL neurons led to presynaptic dysfunction. It is known that synapsins play a role in short-term plasticity of synapses, axon outgrowth, and synaptogenesis (39). All synapsins share a highly homologous short N-terminal domain and central domains, including the A domain, B domain, and the C-domain (40), but variable domains are localized in the C-terminus, including the D-domain and E-domain in Syn Ia, the D-domain and F-domain in Syn Ib, the G-domain, H-domain, and E-domain in Syn IIa, and the G-domain and I-domain in Syn IIb. The function of the H-domain is poorly understood. Our study is the first to report on its role in mediating the direct interaction between Syn2a and Syngr3. Moreover, deletion of domain H in Syn2a leads to a loss in its binding ability with Syngr3. As Syngr3 is distributed exclusively in SVs, our data suggest that Syngr3 as a Syn2a interactor plays a role in presynaptic orchestration during fear extinction. To date, the detailed physiological functions of Syngr3 remain unclear; our study suggests that the binding of Syngr3 with Syn2a is able to disrupt the presynaptic release process. Another independent study reveals that the interaction of Syngr3 with Tau mediates Tau-induced presynaptic defects, such as reduced synaptic vesicle mobility, suppressed SV recruitment for release, and reduced neurotransmission (41). And genetic knockdown of Syngr3 rescued Tau-induced presynaptic defects without inducing additional toxicity (41). We further identified the unique binding sites in Syngr3 that are responsible for the binding with Syn2a. Moreover, we provided 2 strategies (BBB-permeable peptide and ritonavir injection) to block the interaction of Syn2a and Syngr3, and confirmed their effect in the correction of abnormalities in the presynaptic disorder and extinction deficits in the mouse model. We believe that our work paves the way for the specific targeting of the presynaptic functions of Syn2a-Syngr3, both to selectively evaluate the contribution of this pathway to disease progression in animal models, as well as for providing future therapeutic approaches by targeting synaptic dysfunction in PTSD.

Further, by applying the molecular docking strategy, we identified that ritonavir can disrupt the direct binding of Syn2a with Syngr3. We deduced that ritonavir may interact with AA91–99 of Syngr3 through several H-bonds, Van der Waals, pi-cation, and pi-alkyl. Ritonavir is a Cytochrome P450 3A4 (CYP3A4) protease inhibitor that has been used primarily to treat HIV/AIDS (42), with suggested applications in other diseases. For example, it has been shown that ritonavir in combination with metformin inhibits cell proliferation in multiple myeloma and chronic lymphocytic leukemia (43). Recently, the FDA authorized the combination of nimatravir and ritonavir to treat COVID-19 (44). In a mouse model of DYT-TOR1A dystonia, ritonavir has been shown to ameliorate protein mislocalization and restore brain abnormalities (45). In primary hippocampal cultures, administration of ritonavir effectively increased cell survival and blocked the oxidative stress induced by 4-Hydroxynonenal (4-HNE), a lipid-soluble aldehydic product of membrane peroxidation (46). In PC12 cell extracts, ritonavir could inhibit the calcium-activated protease, calpain, which mediated the tissue injury in PTSD (47). As the expression of CYP3A4 in the brain is very limited, it may not be involved in the effect of ritonavir on fear extinction. Here, we found that ritonavir can effectively block the binding affinity of Syn2a with Syngr3 both in vitro and in vivo. Ritonavir can cross the mammalian brain barrier and accumulate within the CNS, such as in the brain parenchyma, CSF, and choroid plexus (48), and we further demonstrated that ritonavir could improve fear extinction in Syn2a-E mice, a PTSD mouse model.

Taken together, in this study we demonstrate that the upregulation of Syn2a binding with AA91–99 of Syngr3 via its H-domain disrupts the presynaptic orchestration in the IL-BLA inhibitory circuit, which leads to the impairment of fear memory extinction. Blocking the physical interaction of Syn2a with Syngr3 by a mimic peptide or ritonavir was able to reverse these abnormalities.

## Methods

### Experimental animals.

Male C57BL/6J mice at 7–8 weeks old were acquired from the National Resource Center of Model Mice (Nanjing, China). The Syn2a-E mouse was generated using a CRISPR/Cas9 system as previously described (29). The mutant mice were of the C57BL/6J genetic background. The animals were housed (4 per cage) in a temperature-controlled room (22 ± 2°C) on a 12-hour light/dark cycle, and given food and water. They were bred in the Experimental Animal Central of Tongji Medical College, Huazhong University of Science and Technology.

### Fear conditioning/extinction paradigm.

We utilized an auditory fear conditioning/extinction paradigm, which involved auditory fear conditioning, extinction training, and extinction testing (49). Freezing behavior was measured as the dependent variable of fear in all training and testing sessions. On day 1, the auditory fear conditioning phase involved the pairing of 5 tone (CS; 30 second, 75 dB, 1 kHz) and foot shock (US; 2 second, 0.8 mA) with 60 second intertrial intervals in the context (context A). Day 2, the mice were returned to their home cages for 1 day of rest. Day 3, the extinction training phase involved 14 CS-alone trials with 10 second intertrial intervals in context B. On day 4, the extinction testing phase also consisted of 14 CS-alone trials with 10 second intertrial intervals in context B. The control mice in this study were kept in their home cages without special treatment. The no-extinction mice were only subjected to a pairing of 5 tone and foot shock stimuli but not subjected to extinction. According to previous literature, the term ‘acquisition’ in this context refers to the mean freezing of the first 2 trials of extinction training on the third day of the fear conditioning/extinction paradigm. On the other hand, the ‘retrieval’ refers to the mean freezing of the first 5 trials of extinction testing on the fourth day of the fear extinction paradigm. The ‘extinction’ refers to the mean freezing of all trials of extinction testing on the fourth day of the fear extinction paradigm, which can also reflect the index of extinction retrieval (5, 50, 51).

### Open field.

Each mouse was placed in the open field arena (60 × 60 × 60 cm) from a fixed corner and allowed to move freely for 5 minutes while being monitored by photo-beam detectors. Data was collected using a computer and analyzed using the MED associates’ Activity Monitor Data Analysis software. Between sessions, the maze was cleaned with 75% ethanol and dried with paper towels. The time spent in the center and side square, as well as the total distances traveled in all areas, were analyzed (29).

### EPM.

The EPM is composed of a center area, 2 open arms, and 2 closed arms. The arms are situated 30.5 cm above the ground. At the beginning of the trial, mice were placed in the center area and allowed to explore the apparatus for 5 minutes. The movement traces of the mice were collected and analyzed using tracking software. The time spent in the open and closed arms were measured (29).

### PCA.

The identification of potential genetic targets during fear extinction sessions heavily relies on distinguishing mice with different extinction patterns, such as those that are extinction-resilient or extinction-susceptible. Manually identifying these tendencies from dozens of mice with each possessing 14 test results totaled 560 data points. Additionally, selection and separation of mice into the different extinction groups may suffer from data inconsistency and human bias. The average time of freezing responses has been used to assess the influence of a certain intervention, but this single value evaluation metric may be too coarse to retain valuable information. To address these issues, we employed PCA, which automatically identifies linear combinations of the original test results that explain the most variance. PCA is commonly used to extract patterns from various data sources in biology and medical research, including the analysis of behavior data (52).

For simplicity in visualization, we retained only the first 2 components, which collectively explained 60.1% of the data (34.1% and 26.0%, respectively). The first component consisted of all positive entries and can be viewed as the freezing time component. A higher value for this component indicated longer total freezing time, which makes sense, since extinction-resilient mice tend to have longer freezing responses in all trials. The second component can be regarded as the reversed extinction component. A higher value for this component suggests longer total freezing time during the first 7 trials and shorter freezing time during the last 7 trials, indicating more effective fear extinction.

### Immunofluorescence.

The mice were anesthetized using avertin (2, 2, 2-tribromoethanol, Sigma-Aldrich) in 0.9% saline solution (20 mL/kg) and transcardially perfused with saline and 4% PFA. The brains were collected, embedded in OCT, and sectioned to a thickness of 30 μm. After washing twice with PBS to remove OCT, the slides were incubated for 60 minutes in a solution containing 0.5% Triton X-100 diluted to 5% BSA to block nonspecific staining. The slides were then incubated overnight at 4°C with primary antibodies listed in Supplemental Table 2. After washing 3 times with PBS, the slides were incubated with secondary antibodies (1:1,000) for 1 hour at room temperature. The slides were washed 3 times with PBS and mounted with cover glass. The images were captured using a Zeiss LSM800 Examiner Z1 confocal microscope at the Microstructural Platform of the University and analyzed with Image J software (53).

### Electrophysiological recording of slice-patch clamping.

We performed electrophysiological recordings of slice-patch clamping to investigate the synaptic transmission in individual neurons within the BLA. Mice were euthanized by isoflurane anesthesia followed by decapitation, and the brain was rapidly dissected into ice-cold artificial cerebrospinal fluid (ACSF) containing (in mM): 124 NaCl, 3 KCl, 1.25 NaH2PO4•2H2O, 26 NaHCO3, 1.2 MgCl2•2H2O, 10.0 C6H12O6, 2.0 CaCl2, 212 sucrose, and 10 glucose, saturated with carbogen gas (95% oxygen, 5% carbon dioxide) all purchased from Sigma-Aldrich. We obtained 300 μm coronal brain slices containing BLA using a Vibratome. The slices were then transferred to a holding chamber containing carbogen-saturated ACSF at 32°C for 30 minutes, followed by room temperature for 1 hour before recording. We performed recordings from the BLA in coronal slices located at Bregma, including anterior-posterior (AP):−1.4 mm; medial-lateral (ML):±3.4 mm; and dorsal-ventral (DV):−4.5 mm. sEPSCs were recorded at a holding potential of −70 mV, where there are no net currents through GABAA receptors. sIPSCs were isolated by recording at a holding potential of 0 mV, the reversal potential of AMPA receptor–mediated (AMPAR-mediated) and NMDA–receptor–mediated (NMDAR-mediated) currents. Currents were recorded in 10 second epochs for a total duration of at least 100 seconds per recording. We analyzed the data using Clampfit 10.0 and Sigmaplot 12.5 software.

### Paired-pulse stimulation.

To investigate presynaptic plasticity at inhibitory synapses, we conducted an experiment using paired-pulse stimulation at 0 mV. In order to do this, we added 10 μM CNQX to the perfusate and placed the stimulation electrode in BLA interneurons to record evoked somatic currents. The paired-pulse paradigm involved using an interpulse interval of 50 milliseconds, and we aimed to determine the presynaptic release probability of the recorded synapses. We delivered 5 pairs of stimuli with a 5 second interval between each pair, and measured the peak amplitudes of both EPSCs. Subsequently, we calculated the PPR by dividing the peak amplitude of the second response by the peak amplitude of the first response (29).

### Western blot.

Mice mPFCs were homogenized in lysis buffer with protease inhibitors and ready for Western blot. Western blotting was carried out as described previously (54). The detailed information for all the antibodies was list in Supplemental Table 2. The protein signals were detected using Odyssey Imaging System (LI-COR) and analyzed using Quantity 1 software (Bio-Rad).

### Real-time PCR.

Total RNA was extracted by TRIzol reagent (Ambion) according to the manufacture’s protocol (55). Then, a total of 1 μg RNA was reverse transcribed into cDNA using the Hifair II 1st Strand cDNA Synthesis Kit (Yeasen). Realtime PCR was performed in a Cycler (Bio-Rad). Expression levels of mRNA were quantified using the iTaq Universal SYBR Green Supermix (Bio-Rad) on the real-time PCR detection System (Applied Biosystems). The primer sequences are listed in Supplemental Table 3.

### Cell pickup and quantitative PCR.

Single labeled cells were visualized using fluorescence microscopy and collected using glass capillaries held by a 4-axis micromanipulator under bright-field optics. The collected cells were then transferred to tubes containing 3 μL of lysis buffer. Subsequently, 10 cells labeled with a fluorescent marker were randomly chosen from each animal. The RNA from each cell was amplified and subjected to quantitative PCR.

### Virus microinjection.

For stereotaxic injection, mice were anesthetized with avertin (2, 2, 2-tribromoethanol, Sigma-Aldrich) in 0.9% saline solution (20 mL/kg). Their heads were then fixed in a stereotaxic apparatus (RWD life science, China). The scalp was sterilized with iodophors, and holes were drilled bilaterally. A total of 0.4 μL of virus was microinjected into the mPFC and/or BLA using an automatic microinjection system (Hamilton Company). The virus was injected into both sides of the BLA (AP:−1.4 mm; ML:±3.4 mm; DV:−4.5 mm) and mPFC (AP:+1.9 mm; ML:±0.25 mm; DV:−2.5 mm). The infusion rate was 0.04 μL/min. The AAV2/8-hSyn1-Syn2a-mCherry, AAV2/8-hSyn1-Syn2b-mCherry, AAV2/8-CMV-Syn2aΔH-EGFP, and AAV2/8-CMV-Syn2aΔE-EGFP viruses were purchased from Obio Technology (Shanghai, China). AAV2/9-hSyn1-EGFP viruses and rAAV-hSyn1-DIO-hM3Dq-EGFP were purchased from Brain Case. rAAV- hSyn1-hChR2-EYFP were purchased from Brain Case (Shenzhen, China). rAAV-DIO-hSyn-H2B-EGFP-TVA and rAAV-DIO-hSyn-RVG were purchased from Brainvta (Wuhan, China). CVS-EnvA-ΔG-mCherry-p2A-Flpo was purchased from Brain Case. rAAV-EF1a-fDIO-EGFP and rAAV-EF1a-fDIO-Syn2a-EGFP were purchased from Brainvta (Wuhan, China). The lentivirus-packaged sh-Syn2a and the scrambled control were purchased from Obio Technology (Shanghai, China). Animals were used for detection 4 weeks after the injection. The injection sites were confirmed upon sacrificing the mice, and mice with misplaced injections were excluded from data analysis (54). For chemogenetic activation experiments, virus was bilateral injected. GAD67-Cre mice were injected rAAV-hSyn1-DIO-hM3Dq-EGFP in the BLA coordinates described above with 250 nL of virus, whereas AAV2/8-hSyn1-Syn2a-mCherry was injected into mPFC with 400 nL of virus. Virus was injected at a rate of 0.04 μL/min. Viral injection coordinates for the BLA were as follows: AP, −1.4 mm (from bregma); ML, ±3.4 mm; and DV, −4.5 mm (from the surface of brain). Viral coordinates for the mPFC were as follows: AP, +1.9 mm (from bregma); ML, ±0.25 mm; and DV, −2.5 mm (from the surface of the brain). For the chemogenetic assays, clozapine N-oxide (CNO) or PBS was administered 40 minutes before behavioral testing.

### Optogenetic stimulation in vitro.

ChR2-EYFP expression was achieved by bilaterally injecting high titers (over 5 × 1,012 genomic particles/mL) of rAAV-Ef1a-hChR2-EYFP in the IL of EXT-S and EXT-R mice. Following the injections, slices were transferred to a holding chamber containing ACSF with the following composition: 124 mM NaCl, 3 mM KCl, 26 mM NaHCO3, 1.2 mM MgCl2, 1.25 mM NaH2PO4, 10 mM C6H12O6, and 2 mM CaCl2 at pH 7.4 and 305 mOsm, all purchased from Sigma-Aldrich. The slices were first incubated at 32°C for 30 minutes and then maintained at 22°C for 60 minutes. Subsequently, a single slice was transferred to a recording chamber, which was continuously perfused with oxygenated ACSF (2 mL/min) at 22°C. Whole-cell patch-clamp recordings were performed on BLA interneurons in each slice, which were visualized using a fluorescent infrared-phase-contrast (IR-DIC) Axioskop 2FS upright microscope equipped with a Hamamatsu C2400-07E infrared camera. Synaptic currents were induced by stimulating IL-BLA axon terminals using a 473-nm laser (DPSS laser, Anilab) (56).

### Drugs.

P-2A (TAT-RFQQISSVR, Chinese peptide, 10 mg/kg), ritonavir (57) (MCE, 5 mg/kg), and the scrambled peptide or vehicles were injected intraperitoneally into mice for 4 days (1 injection per day). Experiments were conducted 12 hours after the last intraperitoneal injection.

### 3D reconstruction.

The 3D images were 3D rendered by Imaris ×64 8.4.1 (Bitplane Software) (58). 3D images were acquired on an Nikon Instruments using a 60 × 1.49 NA lens and index-matched immersion oil. Samples were imaged with a Nikon Plan Apo TIRF lens (NA 1.49, oil immersion) and an Andor DU-897X-5254 camera. Set the z-stack z-step size to 0.120 m as required. 3D images were processed, reconstructed, and analyzed using the Imaris × 64 8.4.1 software. The Wiener and apodization filter parameters were kept constant in all image reconstructions.

### Primary neural culture and transfection and FM4-64 experiment.

The hippocampus was quickly dissected and digested in 0.25% trypsin (Invitrogen) at 37°C for 15 minutes. The tissues were triturated, and the cells were plated on poly D-lysine-coated coverslips or dishes. The cultures were maintained in Neurobasal medium supplemented with 2% B-27 and 1% Glutamax (all from Invitrogen) in a humidified incubator at 37°C with 5% CO_2_. Half of the medium was changed every 4 days. The neurons were transfected with the indicated plasmids using Lipofectamine 3000 (Invitrogen) at 7 days in vitro. FM4-64 experiments were performed on a Zeiss LSM 780 confocal microscope with a 20× objective lens. Briefly, neurons at 14 DIV were loaded with 10 μM FM4-64 in a solution containing 45 mM K+ for 1 minute and then washed with a solution containing 3 mM K+ for 15 minutes. Neurons were then subjected to destaining in a 90 mM K+ solution for 2 minutes, and time-lapse recording was performed (59).

### Cell culture, transfection and Co-IP.

HEK293T cells (ATCC, CRL-3216) were cultured in DMEM supplemented with 10% FBS (MIKX Co., Ltd). Transfections were carried out using Lipofectamine 3000 (Invitrogen) following the manufacturer’s instructions. After 48–72 hours of transfection, cells were harvested and subjected to different assays. In brief, tissues or cells were collected and lysed in RIPA buffer (Beyotime, P0013). After quantification using the BCA protein assay kit (Thermo Fisher Scientific), 1 mg of total extracted protein was incubated with 2 μg of antibodies overnight. Normal rabbit or mouse IgG used as a negative IP control. Then the mixtures were incubated with protein A/G agarose beads for another 4 hours, and washed at least 3 times, followed by boiled for 10 minutes in SDS sample buffer (Bio-Rad). Lysates for an input control were also treated with the equal volume of SDS buffer. The detailed information for all the antibodies were list in Supplemental Table 2 (29).

### Statistics.

The results of statistical analyses were performed using GraphPad Prism (GraphPad Software Inc.). The data are presented as the mean ± SEM. Unpaired or paired 2-tailed *t* tests were used for single comparisons, and 1-way or 2-way ANOVAs followed by Bonferroni post hoc tests were used to make the single-variable comparisons. *P* < 0.05 was considered statistically significant.

### Study approval.

All animal experiments were conducted in accordance with the “Policies on the Use of Animals and Humans in Neuroscience Research,” which were revised and approved by the Society for Neuroscience in 1995, and were approved by the Animal Care and Use Committee of Tongji Medical College.

### Data availability.

Values for all data points in graphs are reported in the Supporting Data Values file.

## Author contributions

LQZ and DL initiated and designed the study. LQZ, YML, ZYG, HYM, XL, and DL supervised the study. XYS, JZ, HZH, LZ and SDL performed the molecular biological experiments and animal experiments. CT and ZQL analyzed the data. XH and SPW performed the electrophysiological recording. XYS carried out 3D reconstruction. ZYG, HYM, XL, and YML provided constructive suggestions. LQZ, XYS wrote the manuscript.

## Supplementary Material

Supplemental data

Unedited blot and gel images

Supporting data values

## Version 1 01/04/2024

In-Press Preview

## Version 2 02/15/2024

Electronic publication
