# Supplementary material for: The interaction of Synapsin 2a and Synaptogyrin-3 regulates fear extinction in mice
Source: J Clin Invest. 2024 Jan 4;134(4):e172802. doi: 10.1172/JCI172802 (PMC10866652; doi:10.1172/JCI172802)
Supplement: Supplemental data [file jci-134-172802-s099.pdf]

Supplemental Figure 1-12

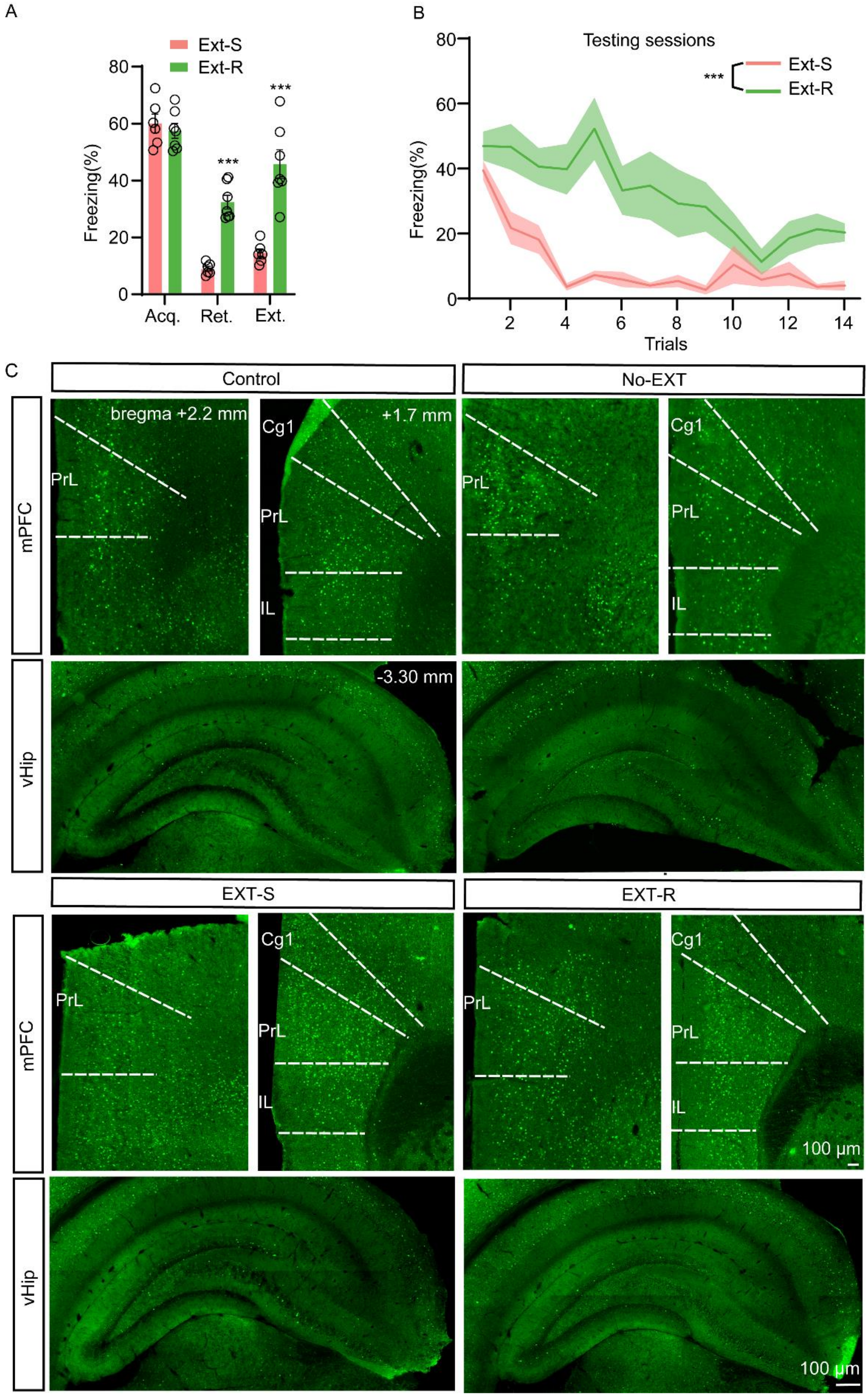

Supplemental Figure 1: Expression profiles of c-Fos in mPFC and ventral hippocampus of EXT-R and EXT-S mice. (**A**) Average freezing response for all trials during the acquisition (Acq.), retrieval (Ret.) and extinction (Ext.) of EXT-S and EXT-R mice (n = 6-7 per group). The term 'acquisition' in this context refers to the mean freezing of the first 2 trials of extinction training sessions. The 'retrieval' refers to the mean freezing of the first 5 trials of extinction testing sessions. The 'extinction' refers to the mean freezing of all trials of extinction testing sessions. (**B**) Freezing response during the extinction testing sessions of EXT-S and EXT-R mice (n = 6-7 per group). (**C**) Representative confocal images of c-Fos staining in the mPFC (cingulate cortex (Cg1), prelimbic cortex (PrL), infralimbic cortex (IL)) and vHip for control, NO-EXT, EXT-S and EXT-R mice. Statistical analyses among multiple groups were conducted using two-way ANOVA followed by Bonferroni post-hoc tests (**A**), whereas unpaired 2-tailed t test was conducted for comparing 2 groups (**B**). ***P < 0.001. Values are presented as mean ± SEM.

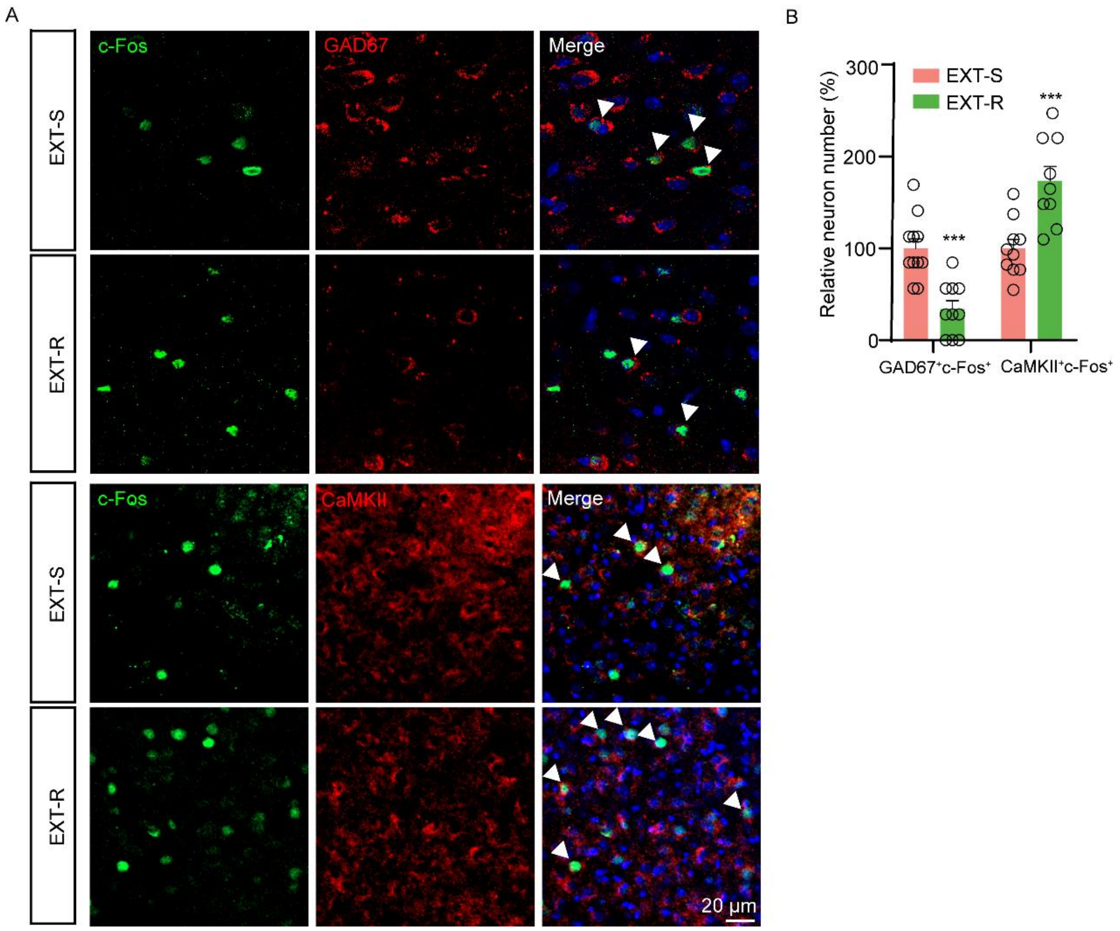

Supplemental Figure 2: Reduced the number of GAD67+/c-Fos+ neurons and increased the number of CaMKII+/c-Fos+ neurons in the BLA of EXT-R mice.

(**A**) Representative immunofluorescence images of CaMKII or GAD67 (red) and c-Fos (green) in the BLA of EXT-S and EXT-R mice. The nucleus was visualized by DAPI (blue). (**B**) Quantification of the percentage of CaMKII$^+$ cells and GAD67$^+$ cells in the c-Fos$^+$ population (n = 3 per group). Statistical analyses among multiple groups were conducted using two-way ANOVA followed by Bonferroni post-hoc tests (**B**). ***P < 0.001. Values are presented as mean ± SEM.

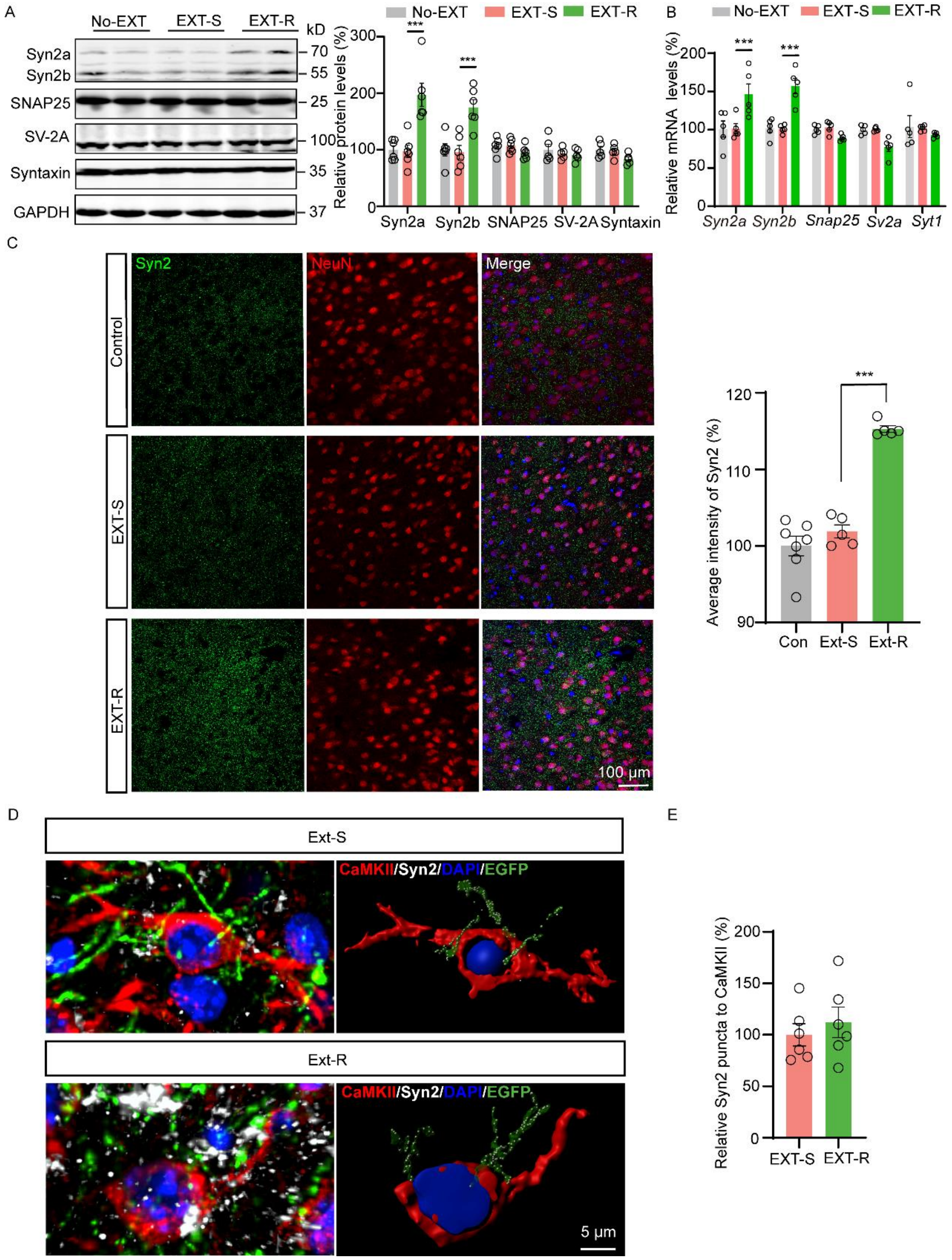

Supplemental Figure 3: Abnormal upregulation of Syn2 in the presynapse of IL-BLA circuit in EXT-R mice. (A) Representative western blots (left) and quantification (right) of the protein levels of partial presynaptic proteins in NO-EXT, EXT-S and EXT-R mice (n = 3 independent experiments). (**B**) qPCR analysis of the

mRNA levels of partial presynaptic proteins in NO-EXT, EXT-S and EXT-R mice (n = 3 per group, normalized to control). (**C**) Representative confocal images of Syn2 (green) and NeuN (red) staining in the IL (left) and quantification (right) of Syn2 intensity for control, EXT-S and EXT-R mice (Slices from 3 mice per group). (**D**) Mice were injected with AAV2/9-hSyn1-EGFP in the IL, and BLA slices were prepared from EXT-S and EXT-R mice for immunostaining with anti-CaMKII (red) and anti-Syn2 (white) antibodies. The nucleus was visualized by DAPI (blue). Representative images of triple immunofluorescence (left) followed by 3D reconstruction (right) were shown. (**E**) Relative average intensity of Syn2 puncta that overlap with green signals from IL to $CaMKII^+$ (upper) cells (n = 6, normalized to control). Statistical analyses among multiple groups were conducted using one-way ANOVA followed by Bonferroni post-hoc tests (**A, B, D**). ***P < 0.001. Values are presented as mean ± SEM.

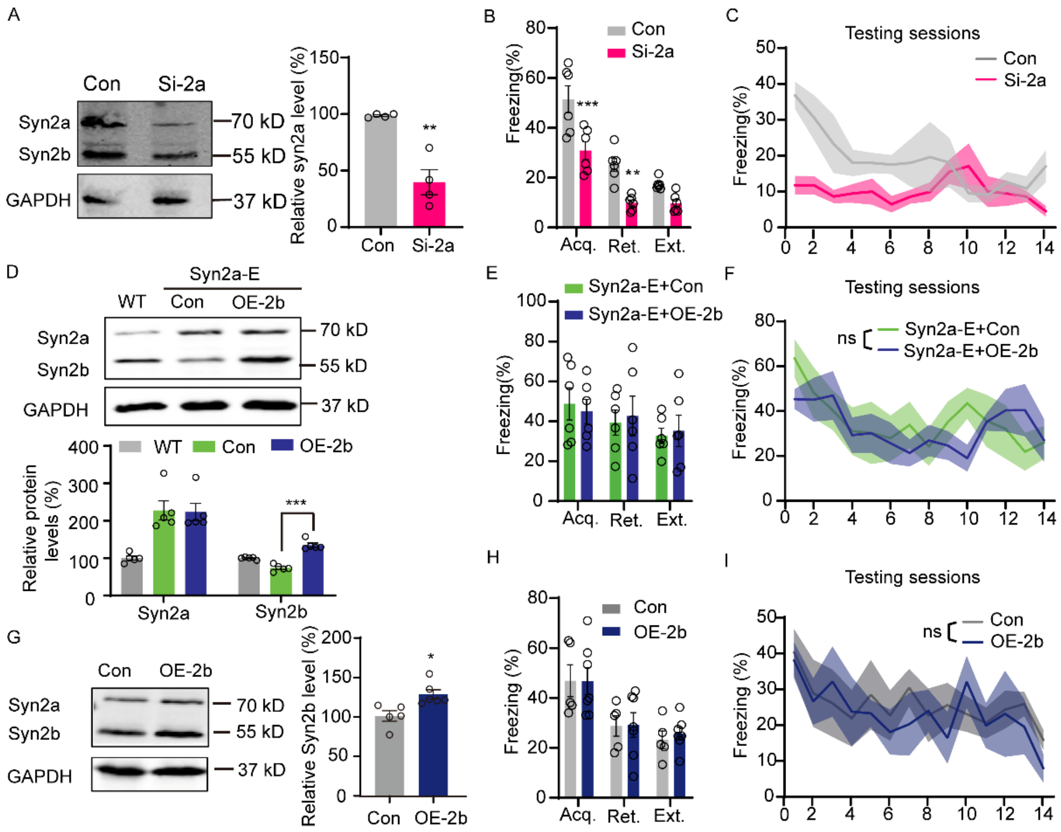

Supplemental Figure 4: Syn2a but not Syn2b is implicated in the extinction of fear memory. (**A**) Representative blots of Syn2a and Syn2b proteins from mPFC homogenates from control or AAV-si-Syn2a virus infected mice (left), and quantification analysis (right) (n = 3 per group). (**B**) Average freezing response for all trials during fear acquisition, extinction retrieval and extinction of control or AAV-si-Syn2a virus infected mice (n = 6 per group). (**C**) Freezing response during the extinction testing sessions of control or AAV-si-Syn2a virus infected mice (n = 6 per group). (**D**) Representative blots (upper) of Syn2a and Syn2b proteins from mPFC homogenates in wild type (WT), AAV2/8-hsyn1-Syn2b-mCherry or control virus infected Syn2a-E mice. Quantification (lower) of relative expression of Syn2a/b (n = 3 per group). (**E**) Average freezing response for all trials during fear acquisition, extinction retrieval and extinction of control and AAV-Syn2b groups in Syn2a-E mice

(n = 6 per group). (**F**) Freezing response during the extinction testing sessions of control and AAV-Syn2b groups in Syn2a-E mice (n = 6 per group). (**G**) Representative blots of Syn2a and Syn2b proteins from mPFC homogenates in control or OE-2b mice (left). Quantification of relative expression of Syn2b (right) (n = 3 independent experiments). (**H**) Average freezing response for all trials during fear acquisition, extinction retrieval and extinction of OE-2b and control mice (n = 5-7 per group). (**I**) Freezing response during the extinction testing sessions of OE-2b and control mice (n = 5-7 per group). Statistical analyses among multiple groups were conducted using one-way (**D**) and two-way (**B, E, H**) ANOVA followed by Bonferroni post-hoc tests, whereas unpaired 2-tailed t test was conducted for comparing 2 groups (**A, C, F, G, I**). $^{*}P < 0.05$, $^{**}P < 0.01$, and $^{***}P < 0.001$. Values are presented as mean ± SEM.

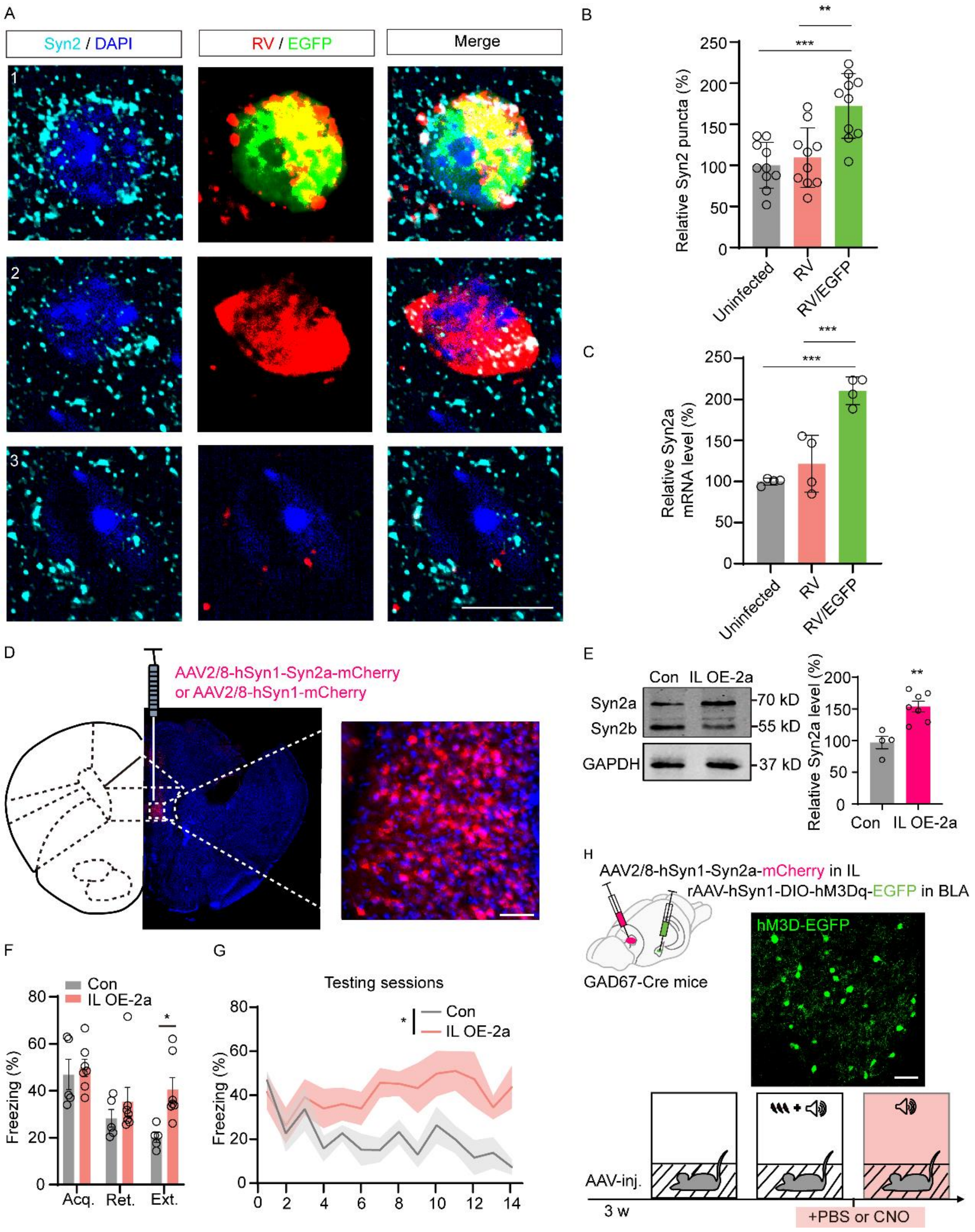

Supplemental Figure 5: Syn2a is implicated in the extinction of fear memory. (**A**) Representative confocal images of Syn2 (cyan) and RV-labeled neurons (red) in the IL that co-localized with the overexpressed Syn2a (yellow). 1, neurons overexpressing Syn2a. 2, RV-labeled neurons. 3, uninfected neurons. Scale bar, 10 µm. (**B**) Quantification of Syn2 intensity in **A**. (**C**) Single cell qPCR analysis of the

mRNA levels in neurons with Syn2a overexpression (RV/EGFP), RV-labeled neurons (RV) and uninfected neurons (Uninfected) (n = 10 per group, normalized to control). (**D**) Representative photomicrographs of injection sites in the IL of AAV2/8-hSyn1-Syn2a-mCherry (IL OE-2a) or AAV2/8-hSyn1-mCherry. Scale bar, 100 μm. (**E**) Representative blots of Syn2a and Syn2b proteins from mPFC homogenates in AAV2/8-hSyn1-mCherry (Con) or AAV2/8-syn1-Syn2a-mCherry (IL OE-2a) injected mice (upper), and the quantification data (lower) (n = 3 independent experiments). (**F**) Average freezing responses of all trials during fear acquisition, extinction retrieval and extinction of IL OE-2a and Con mice (n = 5-7 per group). (**G**) Freezing responses during the extinction testing sessions of IL OE-2a and Con mice (n = 5-7 per group). (**H**) Schematic of AAV injections and experimental design. Statistical analyses among multiple groups were conducted using one-way (**B, C**) and two-way (**F**) ANOVA followed by Bonferroni post-hoc tests, whereas unpaired 2-tailed t test was conducted for comparing 2 groups (**E**, **G**). *P < 0.05, **P < 0.01, and ***P < 0.001. Values are presented as mean ± SEM.

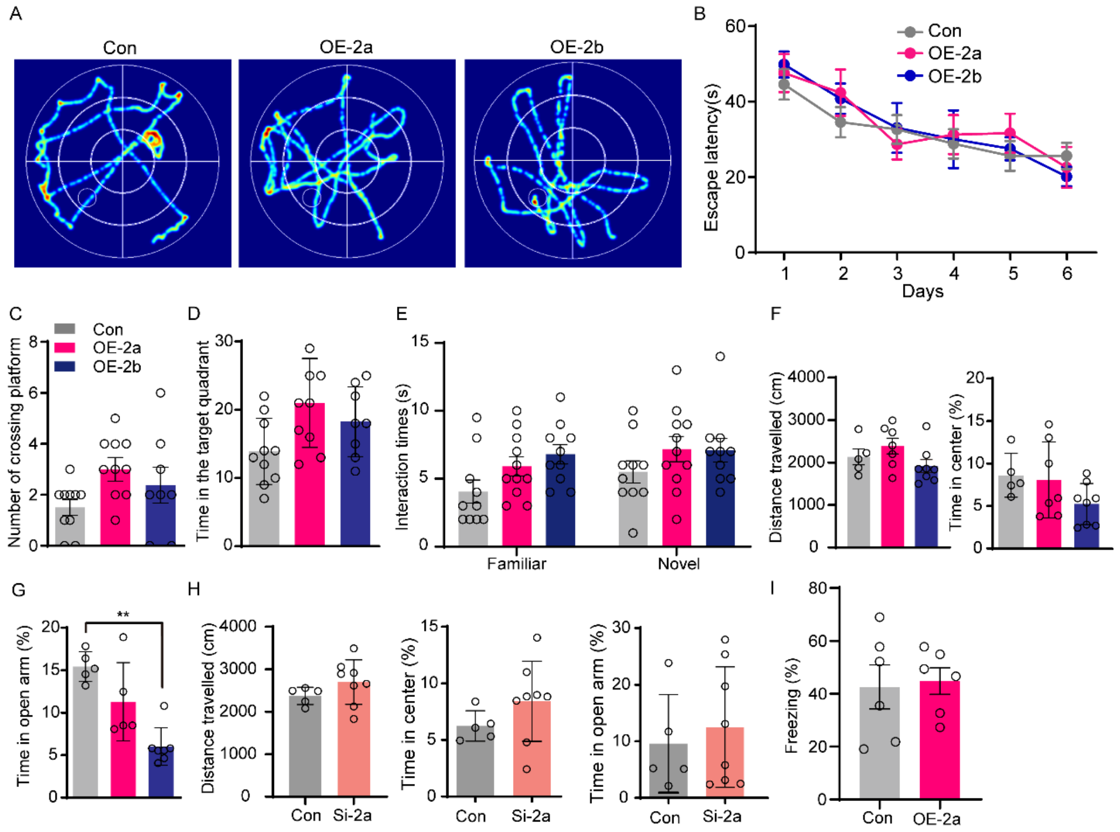

Supplemental Figure 6: Syn2a but not Syn2b is implicated in the extinction of fear memory without emotional abnormalities. (**A**) Representative traces to the hidden platform on day 6 of Morris water maze of control, AAV-Syn2a, AAV-Syn2b virus infected mice. (**B**) Mean escape latency to the platform in the Morris water maze of control, AAV-Syn2a, AAV-Syn2b virus infected mice (n = 8-10 per group). (**C**) Numbers of platform crossing in the Morris water maze of control, AAV-Syn2a, AAV-Syn2b virus infected mice (n = 8-10 per group). (**D**) Time in the target quadrant in the Morris water maze of control, AAV-Syn2a, AAV-Syn2b virus infected mice (n = 8-10 per group). (**E**) The interaction times in the novel object recognition test of control, AAV-Syn2a, AAV-Syn2b virus infected mice (n = 10-11 per group). (**F**) Locomotion and time spent in the center in the open field test of control, AAV-Syn2a, AAV-Syn2b virus infected mice (n = 5-8 per group). (**G**) Time spent in open arm in

the elevated plus maze of control, AAV-Syn2a, AAV-Syn2b virus infected mice (n = 5-7 per group). (**H**) Locomotion and time spent in the center in the open field test of control or AAV-si-Syn2a injected mice (n = 5-8 per group). Time spent in open arm in the elevated plus maze of control or AAV-si-Syn2a injected mice (n = 5-8 per group). (**I**) Average freezing response for all trials during fear acquisition of control or OE-2a virus infected mice using 0.5mA footshock (n = 6 per group). Statistical analyses among multiple groups were conducted using one-way (**B**, **C, D, E, F, G**) ANOVA followed by Bonferroni post-hoc tests, whereas unpaired 2-tailed t test was conducted for comparing 2 groups (**H, I**). $^{**}P < 0.01$. Values are presented as mean ± SEM.

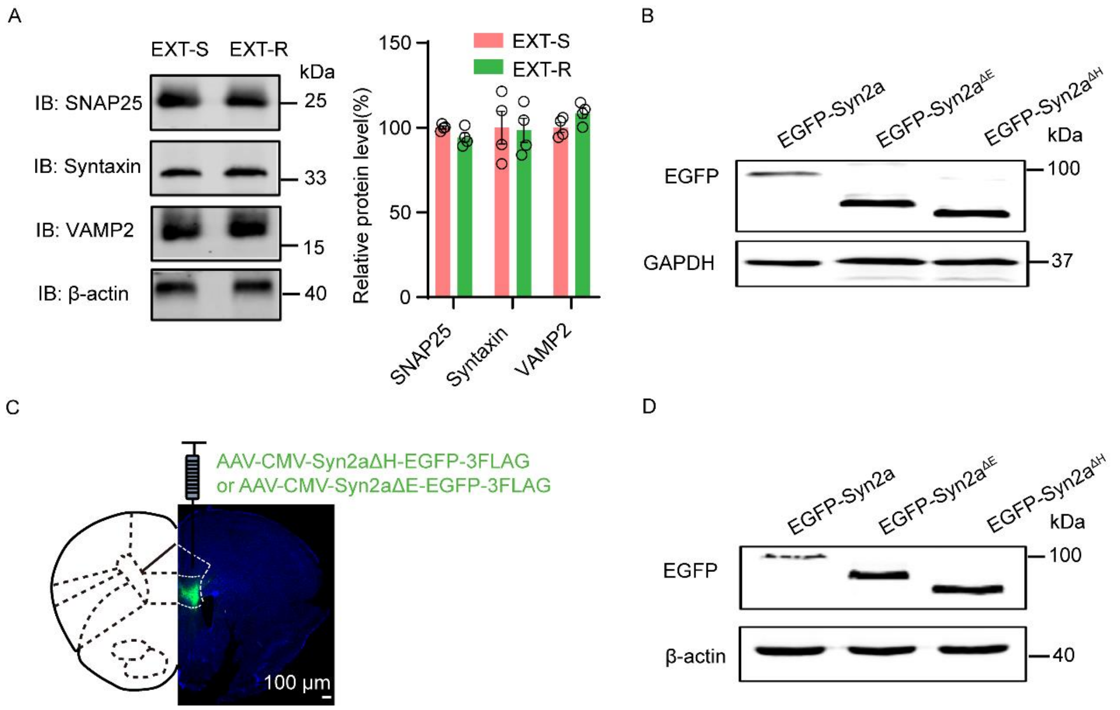

Supplemental Figure 7: Reduced presynaptic SNARE complex formation in the EXT-R mice.

(**A**) Representative western blots (left) and quantification (right) of the protein levels of SNAP25, syntaxin, VAMP2 proteins in EXT-S and EXT-R mice (n = 4 independent experiments). (**B**) The HEK293 cells were transfected EGFP-Syn2a, EGFP-Syn2a-ΔE or EGFP-Syn2a-ΔH plasmid and the cell lysates were collected at 48 hours later. Cell lysates were then subjected to western blot using anti-EGFP antibody. (**C**) Representative photomicrographs of injection sites in the IL with AAV-CMV-Syn2aΔH-EGFP or AAV-CMV-Syn2aΔE-EGFP. (**D**) Representative blots of EGFP proteins from mice infected with mPFC homogenates in EGFP-Syn2a, EGFP-Syn2aΔE and EGFP-Syn2aΔH viruses. Statistical analyses among multiple groups were conducted using two-way (**B**) ANOVA followed by Bonferroni post-hoc tests. Values are presented as mean ± SEM.

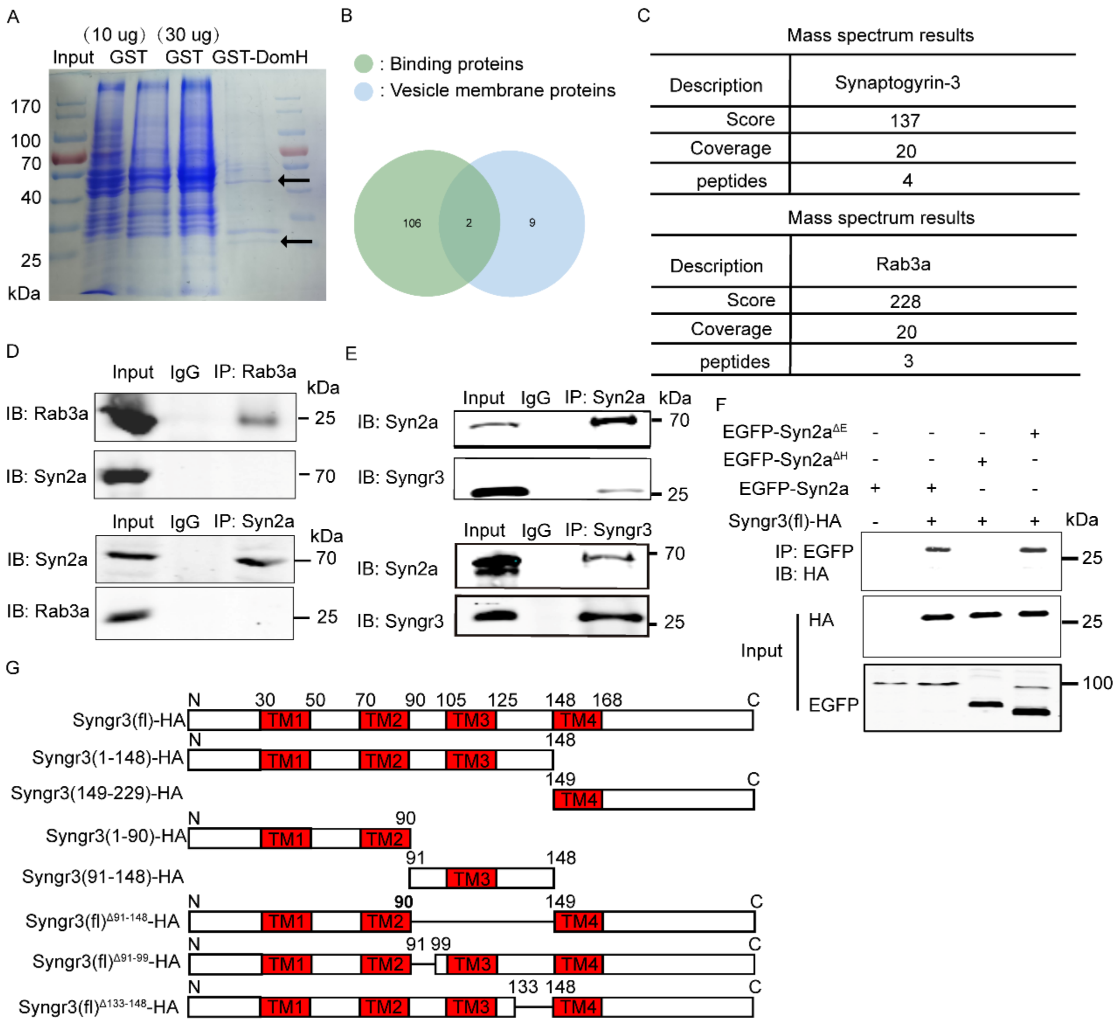

Supplemental Figure 8: Syn2a domain H physically binds with synaptogyrin-3. (**A**) Coomassie stained SDS-PAGE gel of a GST-Syn2a domain H co-immunoprecipitation experiment. Lane 1 is input, lane 2 is 10ug GST immunoprecipitation, lane 3 is 30ug GST immunoprecipitation, lane 4 is GST-Syn2a domain H immunoprecipitation, arrows indicate the two protein bands of 40 kDa and 25 kDa. (**B**) The Venn diagram shows the Syn2a domain H binding proteins measured by mass spectrometry (left circle), vesicle membrane proteins (right circle) and both (overlapping central portion). (**C**) The peptide segments in synaptogyrin-3 and Rab3a that were identified by mass spectrometry. (**D**) Coimmunoprecipitation of Syn2a and Rab3a from mPFC lysates of naïve mice (n = 3 replicates). IP, immunoprecipitation;

IB, immunoblotting. (**E**) Co-immunoprecipitation of Syn2a and Syngr3 from mPFC lysates of naïve mice (n = 3 replicates). (**F**) H293T cells were transiently transfected with the EGFP-Syn2a, EGFP-Syn2a-ΔH, EGFP-Syn2a-ΔE and Syngr3 (fl)-HA plasmids. The cell lysates were collected and immunoprecipitated with an anti-EGFP antibody. Western blots were performed by using anti-HA and anti-EGFP antibodies. (**G**) Illustration of the different Syngr3 mutant constructs. The Syngr3 (fl) protein is referred to as the full length wild type mouse Syngr3 (wt) protein. TM means transmembrane.

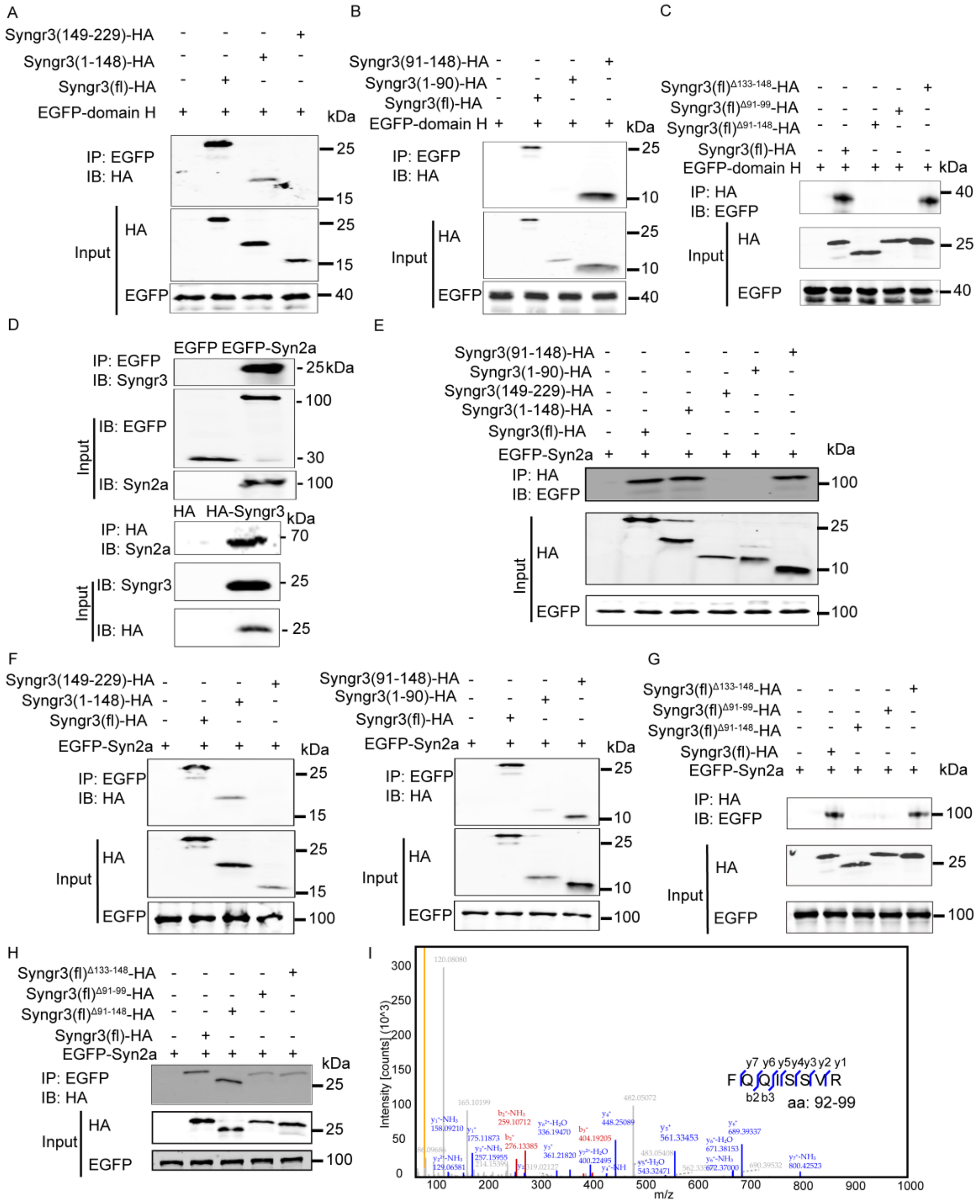

Supplemental Figure 9: The amino acid 91-99 in Syngr3 is essential for its interaction with Syn2a. (**A-C**) H293T cells were transiently transfected with the Syngr3 (fl)-HA, Syngr3 (1-148)-HA, Syngr3 (149-229)-HA, Syngr3 (1-90)-HA, Syngr3 (91-148)-HA, Syngr3 (deletion 91-148)-HA, Syngr3 (deletion 91-99)-HA, Syngr3 (deletion 133-148)-HA and EGFP-domain H plasmids. The cell lysates were collected and immunoprecipitated with an anti-EGFP antibody (**A, B**) and an anti-HA

antibody (**C**). Western blots were performed by using anti-HA and anti-EGFP antibodies. (**D**) H293T cells were transiently transfected with the EGFP-Syn2a or EGFP-C1 plasmids. The cell lysates were collected and immunoprecipitated with an anti-EGFP antibody. Western blots were performed by using anti-Syngr3, anti-Syn2a and anti-EGFP antibodies. H293T cells were transiently transfected with the HA or HA-Syngr3 plasmids. The cell lysates were collected and immunoprecipitated with an anti-HA antibody. Western blots were performed by using anti-Syngr3, anti-Syn2a and anti-HA antibodies. (**E, F**) H293T cells were transiently transfected with the Syngr3 (fl)-HA, Syngr3 (1-148)-HA, Syngr3 (149-229)-HA, Syngr3 (1-90)-HA or Syngr3 (91-148)-HA, and EGFP-Syn2a plasmids. The cell lysates were collected and immunoprecipitated with an anti-HA antibody (**E**) and anti-EGFP antibody (**F**). Western blots were performed by using anti-HA and anti-EGFP antibodies. (**G, H**) H293T cells were transiently transfected with the Syngr3 (fl)-HA, Syngr3 (deletion 91-148)-HA, Syngr3 (deletion 91-99)-HA, Syngr3 (deletion 133-148)-HA, and EGFP-Syn2a plasmids. The cell lysates were collected and immunoprecipitated with an anti-HA antibody (**G**) and anti-EGFP antibody (**H**). Western blots were performed by using anti-HA and anti-EGFP antibodies. (**I**) Identification of peptides in Syngr3 that precipitated by Syn2a by mass spectrometry.

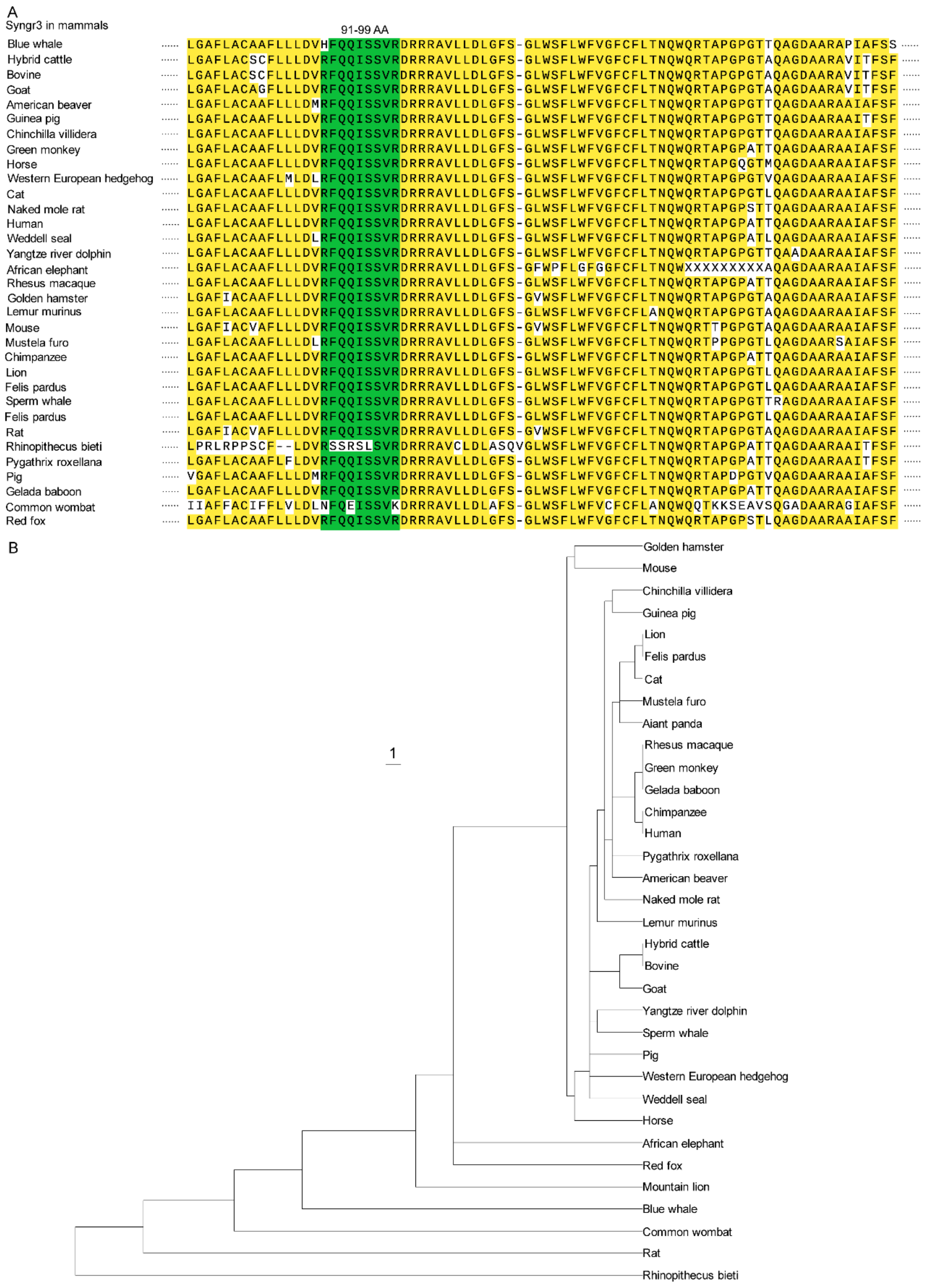

Supplemental Figure 10: Mammalian Syngr3 proteins. (**A**) Alignments of amino acid sequences of Syngr3 proteins from selected mammalian species are shown in single letter code. Identical and different sequences among species are indicated, with

identities being labeled by white background. The 91-99AA region is highlighted in green. (**B**) Phylogenetic tree of Syngr3 in mammals. The scale bar indicates 1 substitution per site and the length of each branch approximates the evolutionary distance.

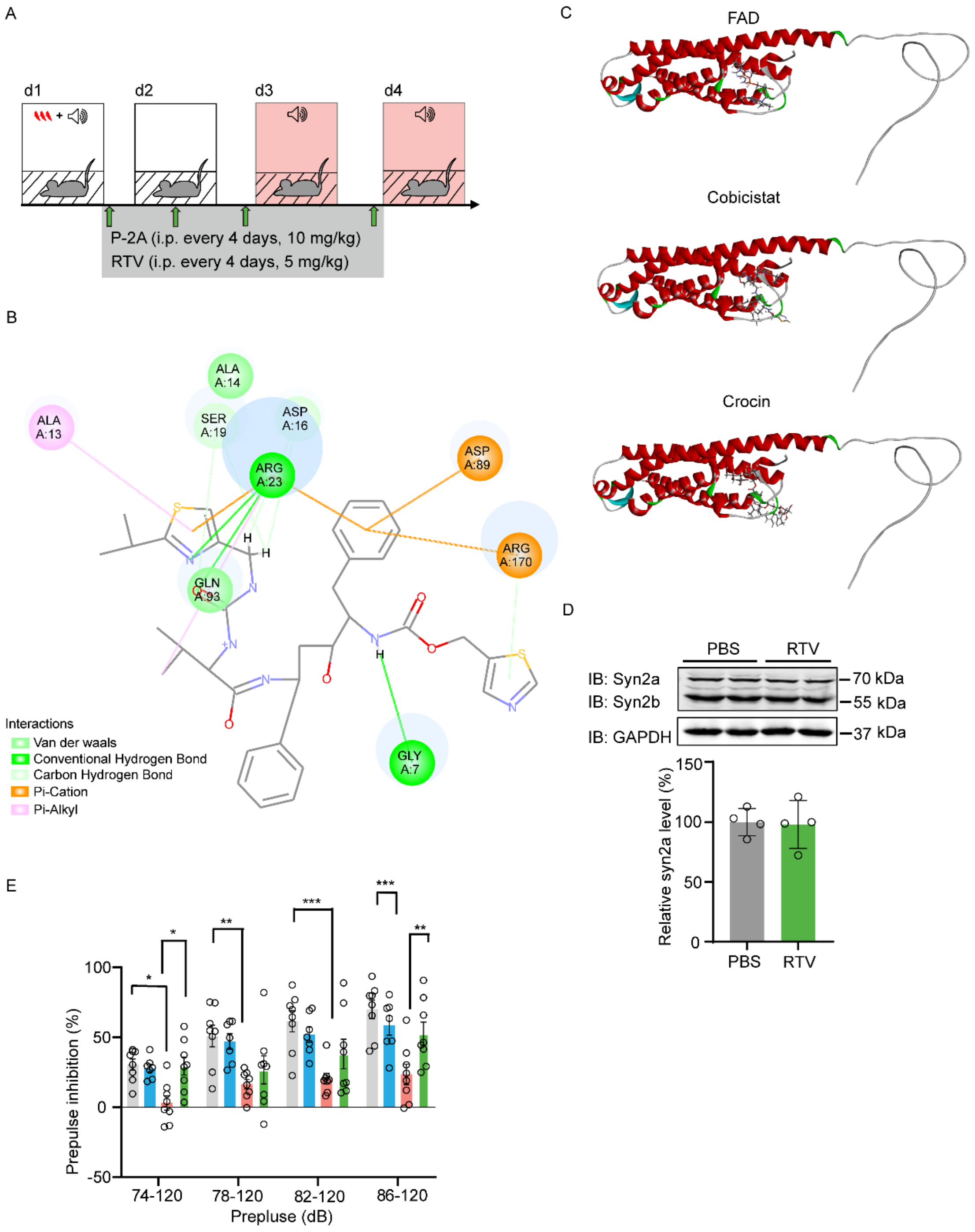

Supplemental Figure 11: Ritonavir can block the Syn2a/Syngr3 interaction. (**A**) A schematic illustration of P-2A and ritonavir administration in mice with fear extinction task. The mice were injected with P-2A or S-2A at the dose of 10 mg/kg for 4 times. The mice were injected with ritonavir or saline at the dose of 5 mg/kg for 4 times. (**B**) Ritonavir is tightly bound onto Syngr3 91-99AA with H-bonds, Van der

Waals, pi-cation and pi-alkyl interactions between them. (**C**) Molecular docking of FAD, Cobicistat, Crocin on Syngr3. Syngr3 is labeled in red while FAD, Cobicistat and Crocin are labeled in grey. (**D**) The mice were injected with P-2A or S-2A at the dose of 10 mg/kg for 4 times. Representative western blots (upper) and quantification (lower) of the protein levels of Syn2a and Syn2b in P-2A and S-2A treated mice (n = 3 replicates). (**E**) Comparisons of percentages of prepulse inhibition of startle responses in WT+PBS, WT+ritonavir, Syn2a-E+PBS, Syn2a-E+ritonavir groups in mice with different startle amplitudes (n = 8). Statistical analyses among multiple groups were conducted using two-way (**E**) ANOVA followed by Bonferroni post-hoc tests, whereas unpaired 2-tailed t test was conducted for comparing 2 groups (**B**). *P < 0.05, **P < 0.01, and ***P < 0.001. Values are presented as mean ± SEM.

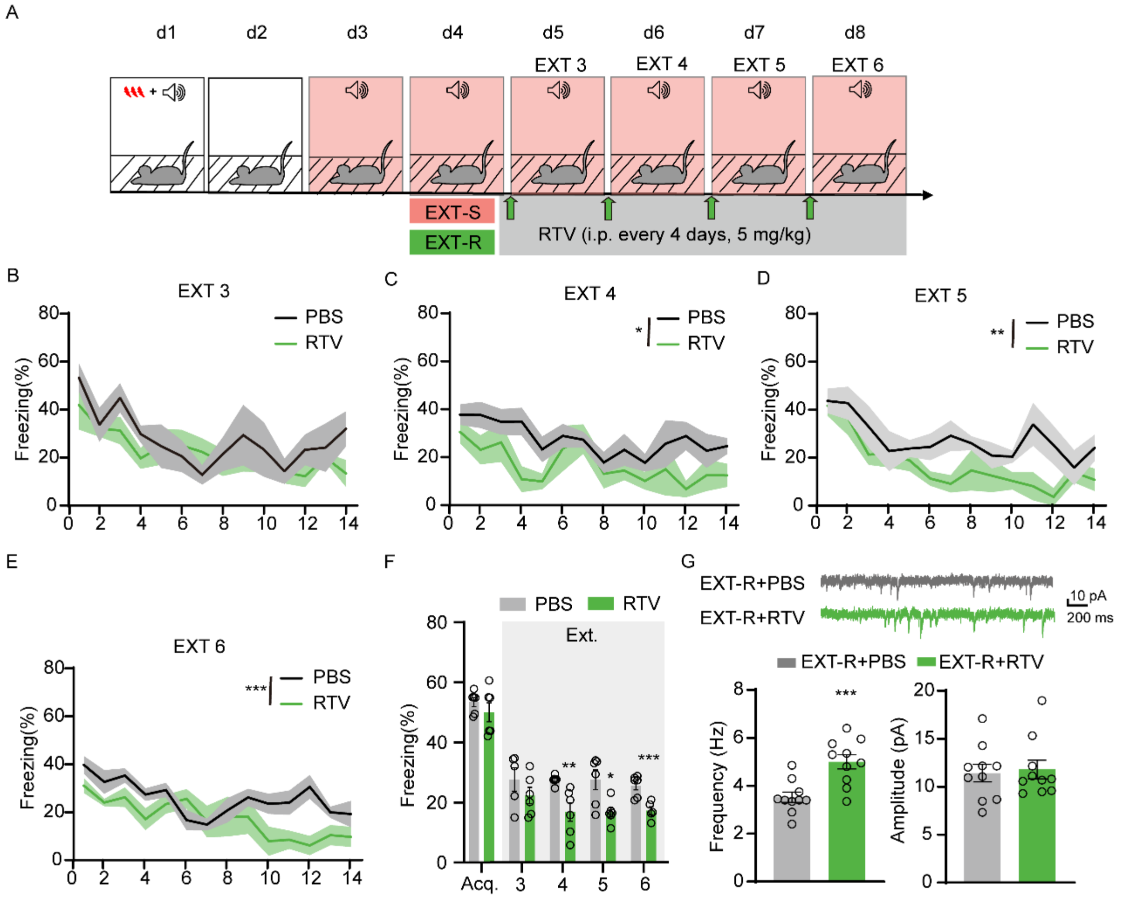

Supplemental Figure 12: Ritonavir facilitates extinction in EXT-R mice. (**A**) A schematic illustration of ritonavir administration in mice with a fear extinction task. The mice were injected with ritonavir at a dose of 5 mg/kg for 4 days. (**B-E**) Freezing responses during the extinction sessions (day 5-8) of EXT-R+PBS and EXT-R+ritonavir mice (n = 6 per group). EXT 3, day 5; EXT 4, day 6; EXT 5, day 7; EXT 6, day 8. (**F**) Average freezing responses of all trials during fear acquisition and extinction of EXT-R+PBS and EXT-R+ritonavir groups of mice (n = 6 per group). (**G**) Representative traces and quantification of the frequency and amplitude of sEPSCs from BLA interneurons in ritonavir or PBS treated EXT-R mice (n = 10 neurons from 3 mice per group). Statistical analyses among multiple groups were conducted using two-way (**F**) ANOVA followed by Bonferroni post-hoc tests, whereas unpaired

2-tailed t test was conducted for comparing 2 groups (**B, C, D, E, G**). *P < 0.05, **P < 0.01, and ***P < 0.001. Values are presented as mean ± SEM.

Tables S1 to S3

table S1. The predicted 4 commercial compounds

Zinc id	Libdock score	Molecule Names	Cat NO.
ZINC000008215434	216.424	FAD	HY-B1654
ZINC000026824305	198.671	Ritonavir	HY-90001
ZINC000245224178	193.412	Crocin	HY-N0697
ZINC000085537014	187.985	Cobicistat	HY-10493

table S2. Antibodies used in current study

Antibody	Catalog number/manufacturer	Dilution
Synapsin II	adi-vas-sv061-e, Enzo	1:1000 for wb<br>1:100 for IP
Synapsin I	ab254349, abcam	1:1000 for wb
SV-2A	ab254351, abcam	1:1000 for wb
VAMP2	13508s, CST	1:1000 for wb<br>1:100 for IP
SNAP25	14903-1-AP, proteintech	1:800 for wb
GAP43	ab75810, abcam	1:1000 for wb
α-synucein	ab138501, abcam	1:1000 for wb
Synaptophysin	ab32127, abcam,	1:1000 for wb
Syntaxin 1α	66437-1-Ig, proteintech	1:1000 for wb
Synaptotagmin-3	12726-1-AP, proteintech,	1:1000 for wb
GAPDH	60004-1-Ig,	1:3000 for wb

	proteintech,	
Synaptogyrin-3	sc-271046, santa cruz	1:000 for wb<br>1:100 for IP
EGFP	ab6556, abcam	1:1000 for wb<br>1:100 for IP
HA	#3724, CST	1:1000 for wb<br>1:100 for IP
Rab3a	15029-1-AP, proteintech,	1:1000 for wb<br>1:50 for IP
c-Fos	226004, Synaptic systems	1:300 for IF
CamKII	ab134041, abcam	1:200 for IF
Parvalbumin (PV)	ab11427, abcam	1:400 for IF
Synapsin 2a	santa cruz, sc-136086	1:100 for IF<br>1:50 for IP
GST	AG768-1, Beyotime	1:1000 for wb<br>1:200 for IP

table S3. Sequences of oligonucleotide primers

Target gene	Species	primer sequence
Snap25 forward	mus	CAACTGGAACGCATTGAGGAA
Snap25 reverse	mus	GGCCACTACTCCATCCTGATTAT
Rab5c forward	mus	TGGTCCTCCGCTTTGTCAAG
Rab5c reverse	mus	TGACCGTTGTATCGTCTAAGCA
Rab5a forward	mus	GCTAATCGAGGAGCAACAAGAC
Rab5a reverse	mus	CCAGGCTTGATTTGCCAACAG
Rab5b forward	mus	GGGAAGTCTAGCCTGGTGTTA
Rab5b reverse	mus	GCTTTCCTGGTATTCATGGAACT
Rab3a forward	mus	GTGGGCAAAACCTCGTTCCT
Rab3a reverse	mus	TCCTCTTGTCGTTGCGGTAGA
Rab3b forward	mus	CCTCCTTCCTTTTCCGCTATG
Rab3b reverse	mus	TCACACGCTTCTCATGGCG
Unc13b forward	mus	TGCTCTGTGTGCGTGTTAAAA
Unc13b reverse	mus	CAGACGACTGATCTCAAACATGA
Stxbp2 forward	mus	AAGGCGGTGGTAGGGGAAA
Stxbp2 reverse	mus	CAACAGGATGACAAGATTCGCA

Vti1a forward	mus	GGATCGCCTACAGTGACGAAG
Vti1a reverse	mus	CAGCCTCTCCGTGTTATCCAG
Dnajc5 forward	mus	ACTTCCGGGGAATCATTATACCA
Dnajc5 reverse	mus	GGGTTATCAGGGTTCTTGTCAG
Synaptophysin forward	mus	CAGTTCCGGGTGGTCAAGG
Synaptophysin reverse	mus	ACTCTCCGTCTTGTTGGCAC
Syt1 forward	mus	CTGTCACCACTGTTGCGAC
Syt1 reverse	mus	GGCAATGGGATTTTATGCAGTTC
Rims1 forward	mus	CCCCCTATGCAAGAACTGCC
Rims1 reverse	mus	CGCCATGTCCCTGACAACA
Snca forward	mus	AAGCCCCTGAATACCTTGATTTG
Snca reverse	mus	GCTGGGATGGTCTTGAGAGAG
Sv-2a forward	mus	GGCTTTCGAGACCGAGCAG
Sv-2a reverse	mus	GACCTTCGGGAATACTCATCCT
Stx1a forward	mus	AGAGATCCGGGGCTTTATTGA
Stx1a reverse	mus	AATGCTCTTTAGCTTGGAGCG
Gap43 forward	mus	TGGTGTCAAGCCGGAAGATAA
Gap43 reverse	mus	GCTGGTGCATCACCCTTCT
Syn1a forward	mus	GGCAAGGACGGAAGGGAT
Syn1a reverse	mus	TTGACCACAAGTTCCACGAT

Syn1b forward	mus	AATGCCTTCAACCTTCCAGA
Syn1b reverse	mus	TTCATTCAGTCGGAGAAGAGG
Syn2a forward	mus	AGATGCCTGCTCTGAAATGTT
Syn2a reverse	mus	ACTCTGTGGCTGTTGGGTG
Syn2b forward	mus	AGACACTGGGAGGGGAGAA
Syn2b reverse	mus	GCATAAGTTTGGGTGAGGG
Gapdh forward	mus	AGGTCGGTGTGAACGGATTTG
Gapdh reverse	mus	TGTAGACCATGTAGTTGAGGTCA
