# Supplementary material for: The interaction of Synapsin 2a and Synaptogyrin-3 regulates fear extinction in mice
Source: J Clin Invest. 2024 Jan 4;134(4):e172802. doi: 10.1172/JCI172802 (PMC10866652; doi:10.1172/JCI172802)

Figure 2A

GAPDH and Tublin are loading controls.

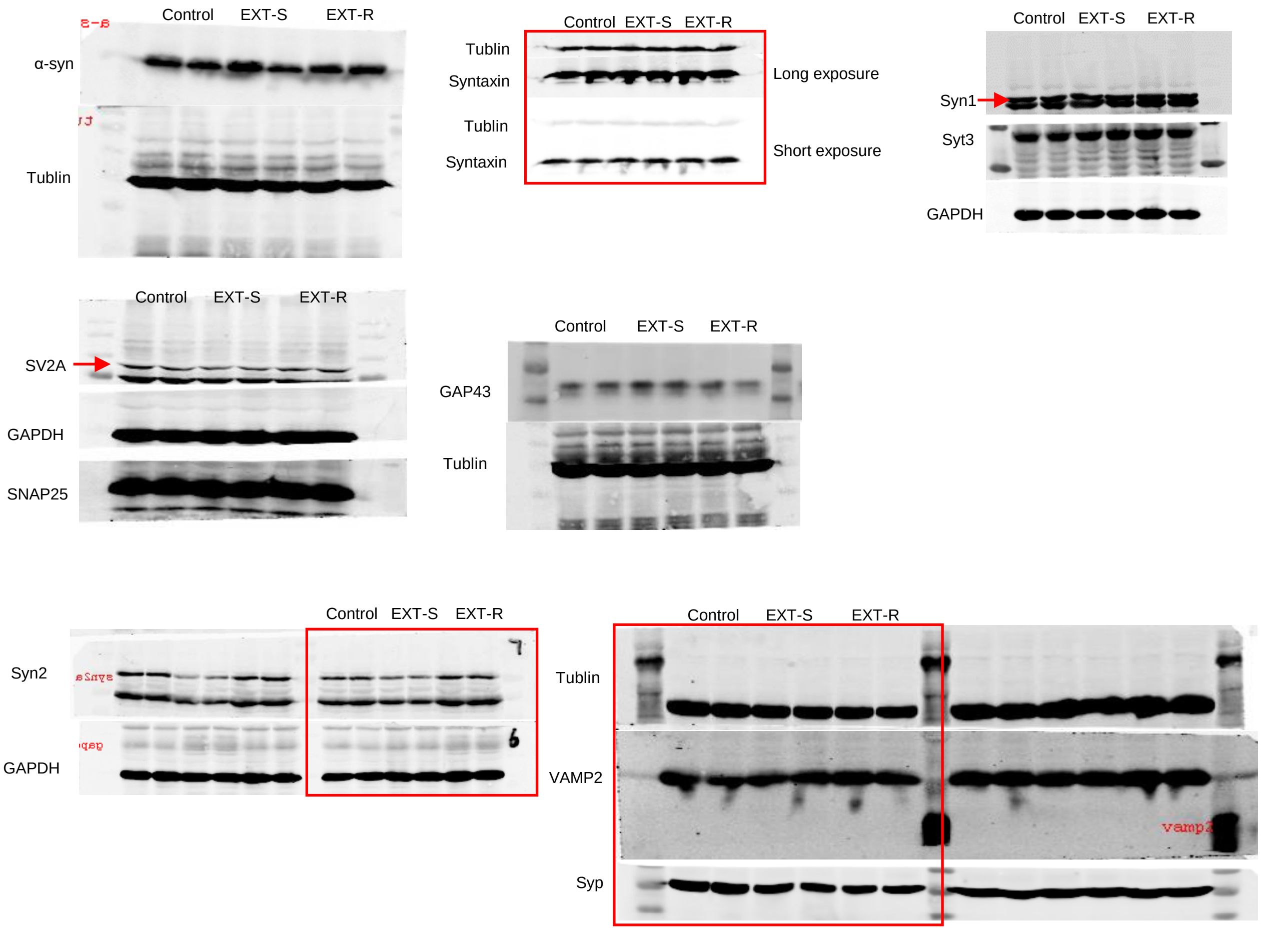

Figure 2D

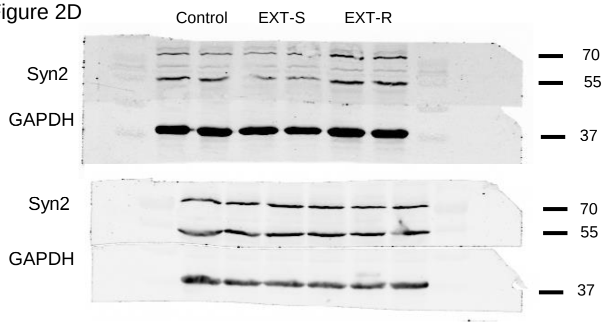

Figure 3A

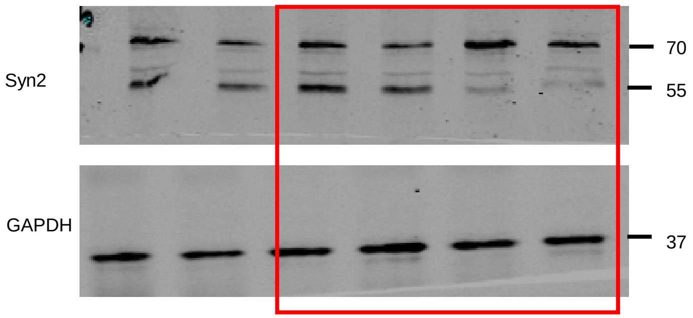

Figure 3I

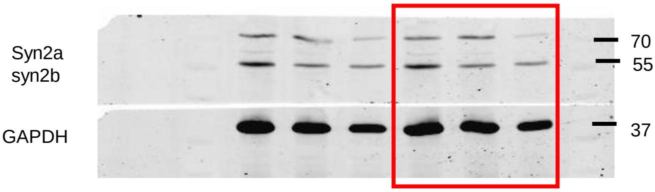

Figure 5A

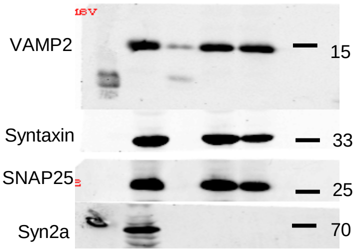

Figure 6A

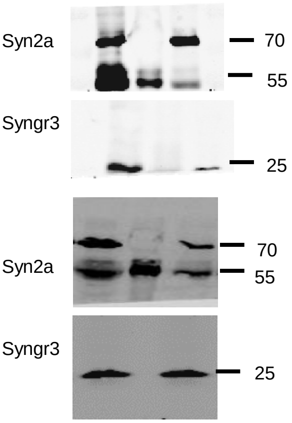

Figure 6B

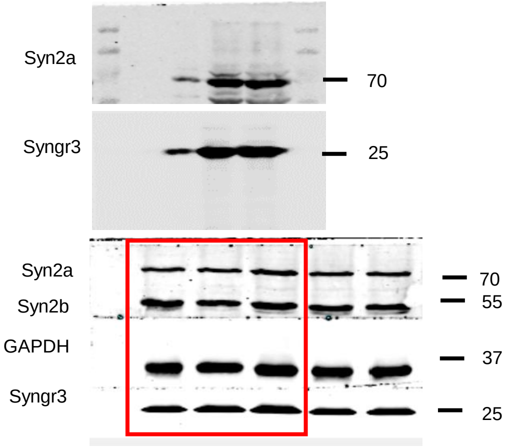

Figure 6C

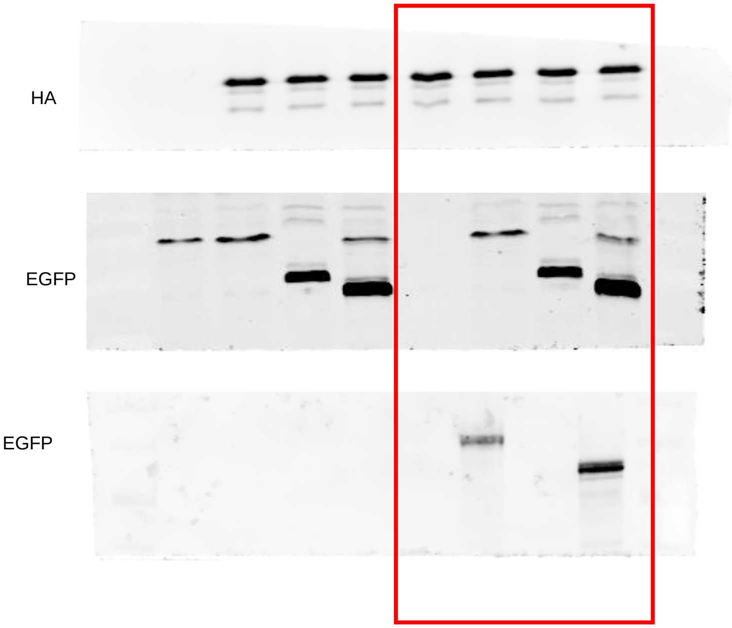

Figure 6E

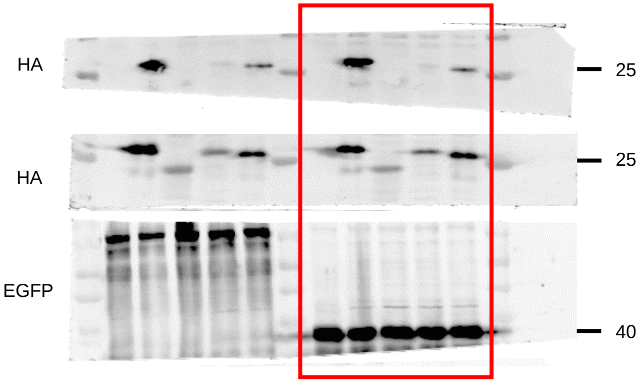

Figure 6D

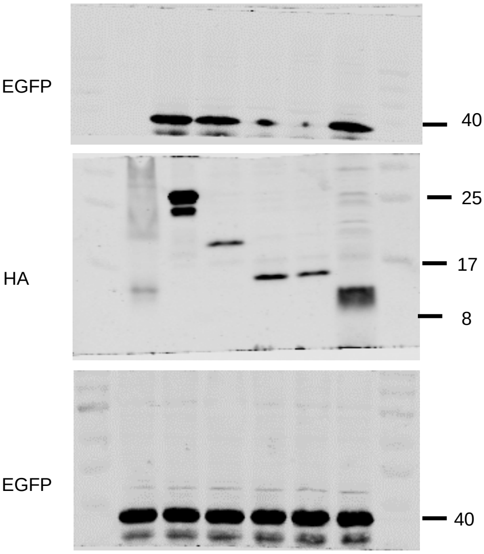

Figure 7B

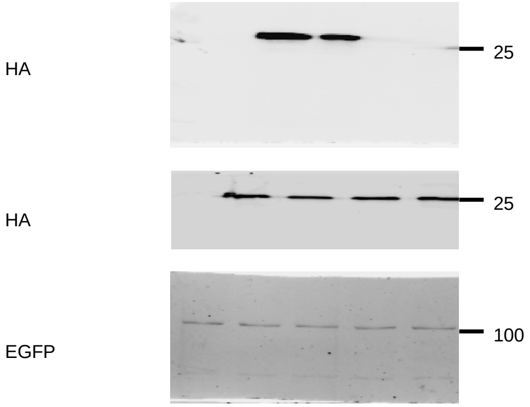

Figure 7C

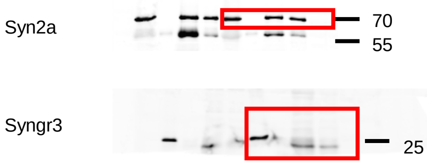

Figure 7D

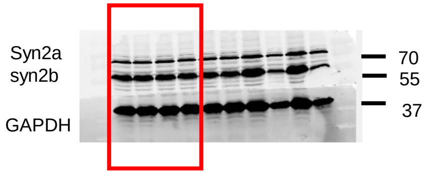

Figure 7E

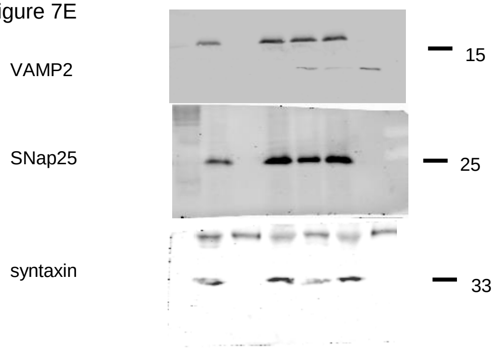

Figure 8A

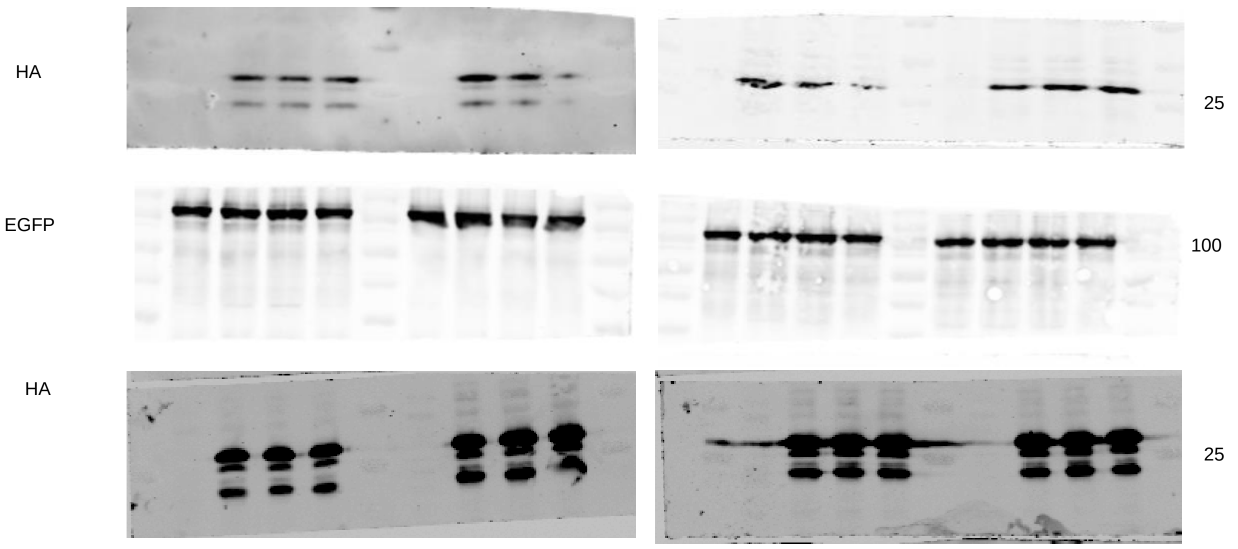

Figure 8C

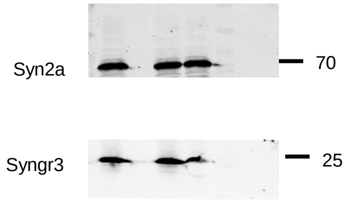

Figure 8D

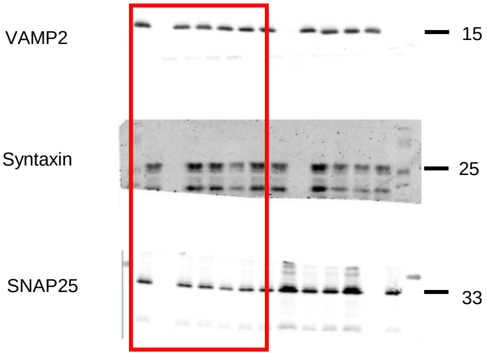

Figure S3A

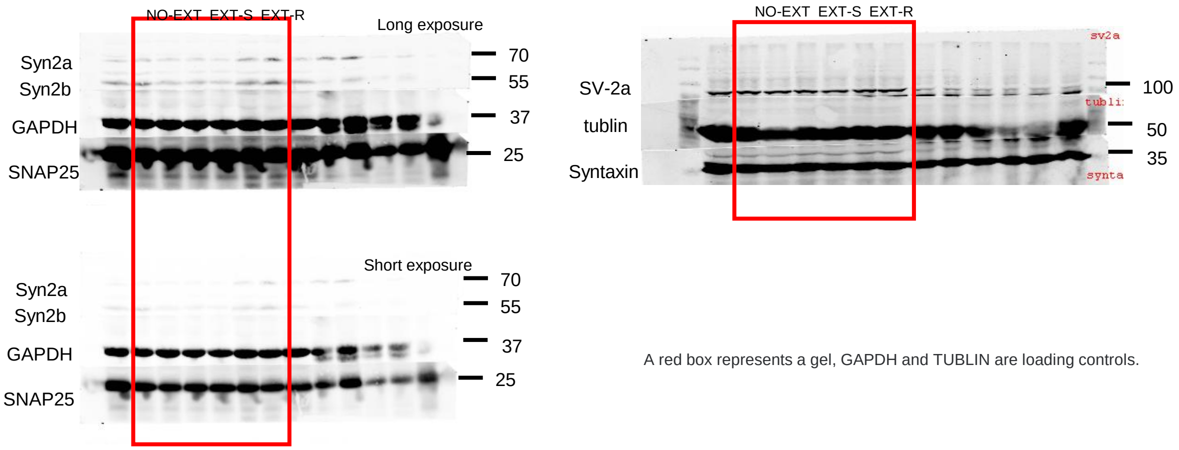

A red box represents a gel, GAPDH and TUBLIN are loading controls.

Figure S4A, S5E

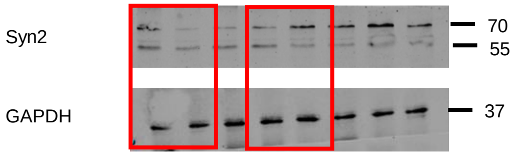

Figure S4G

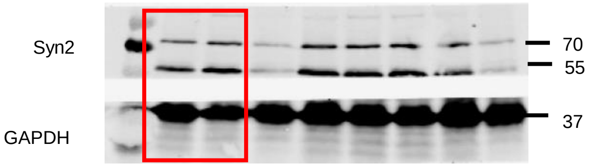

Figure S4D

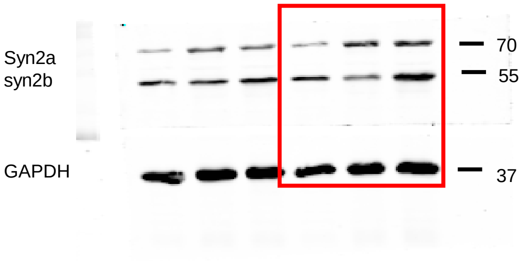

Figure S7A

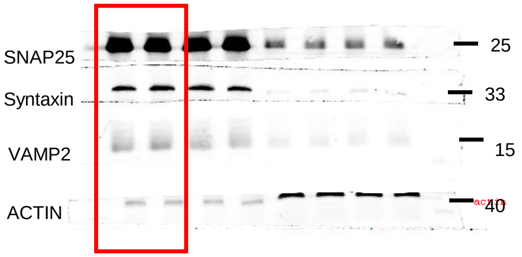

Figure S7B

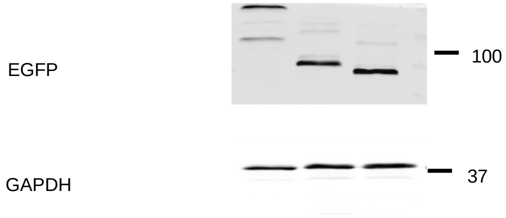

Figure S7D

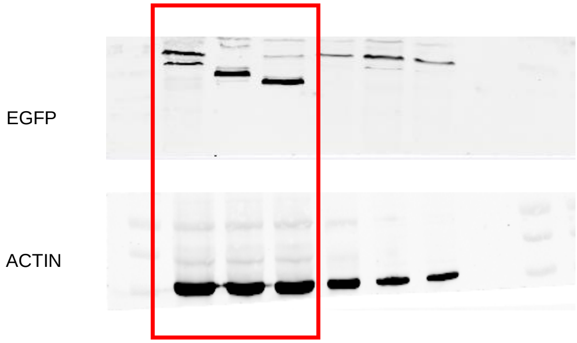

Figure S8D

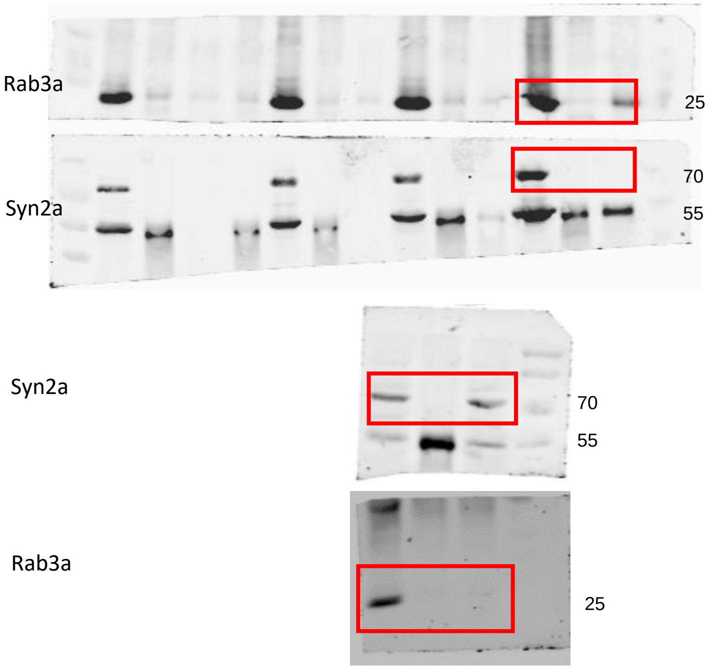

Figure S8E

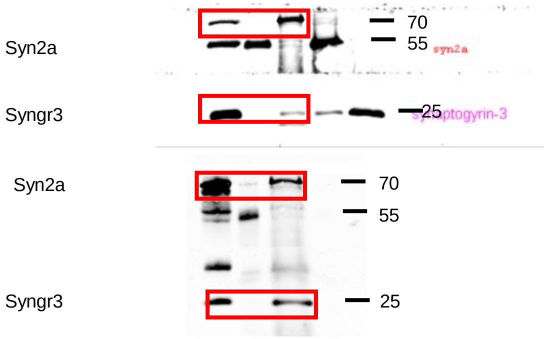

Figure S8F

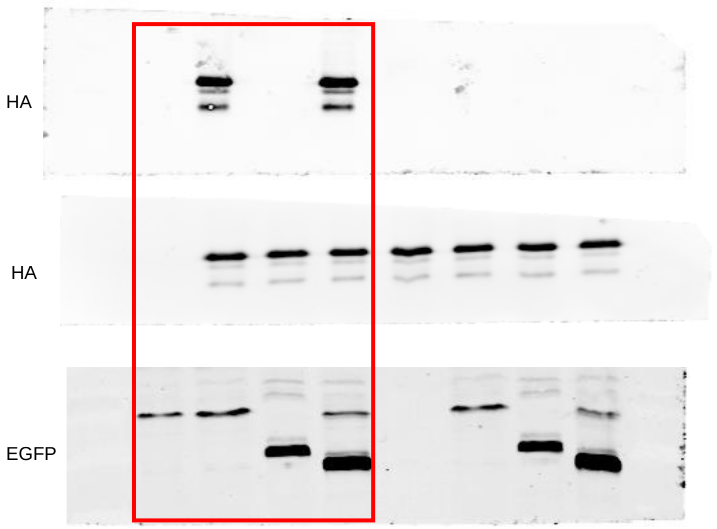

Figure S9A

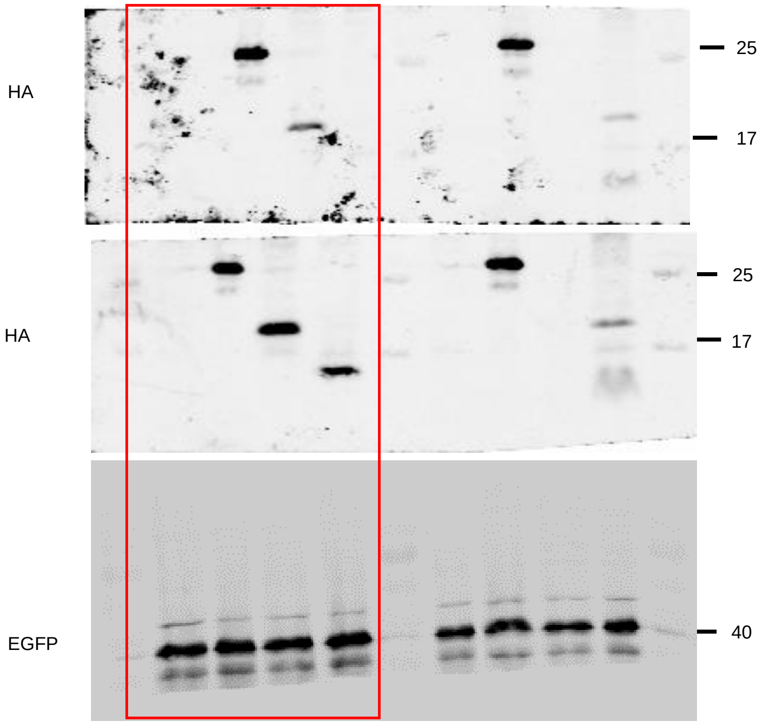

Figure S9B

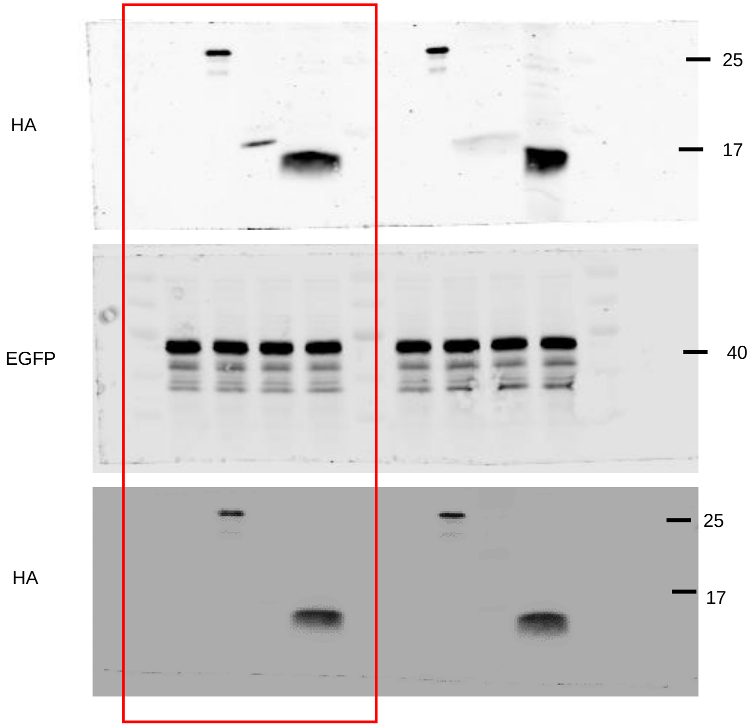

Figure S9C

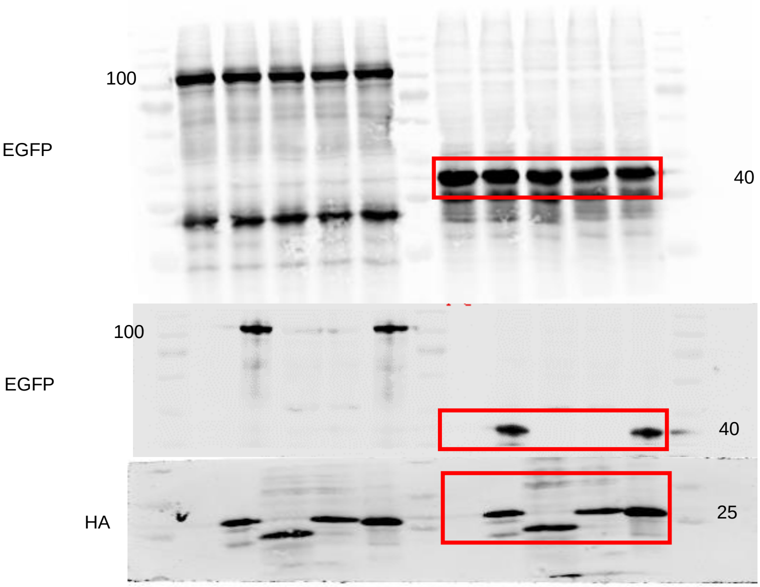

Figure S9D

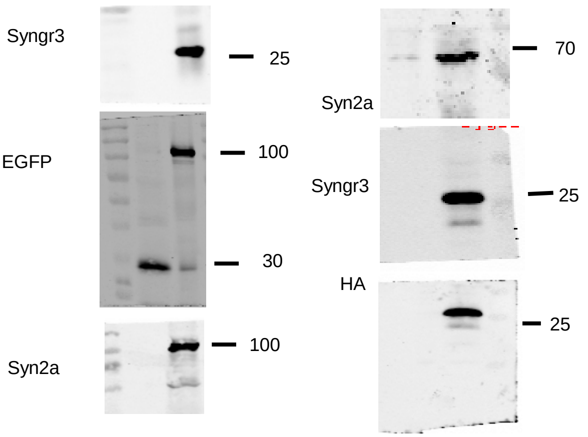

Figure S9E

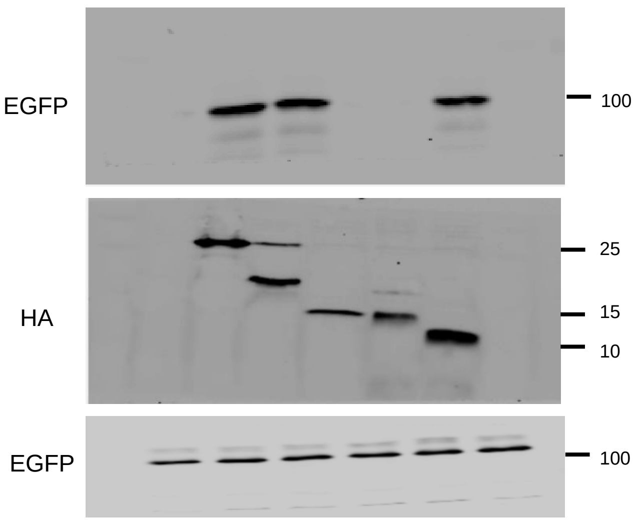

Figure S9F

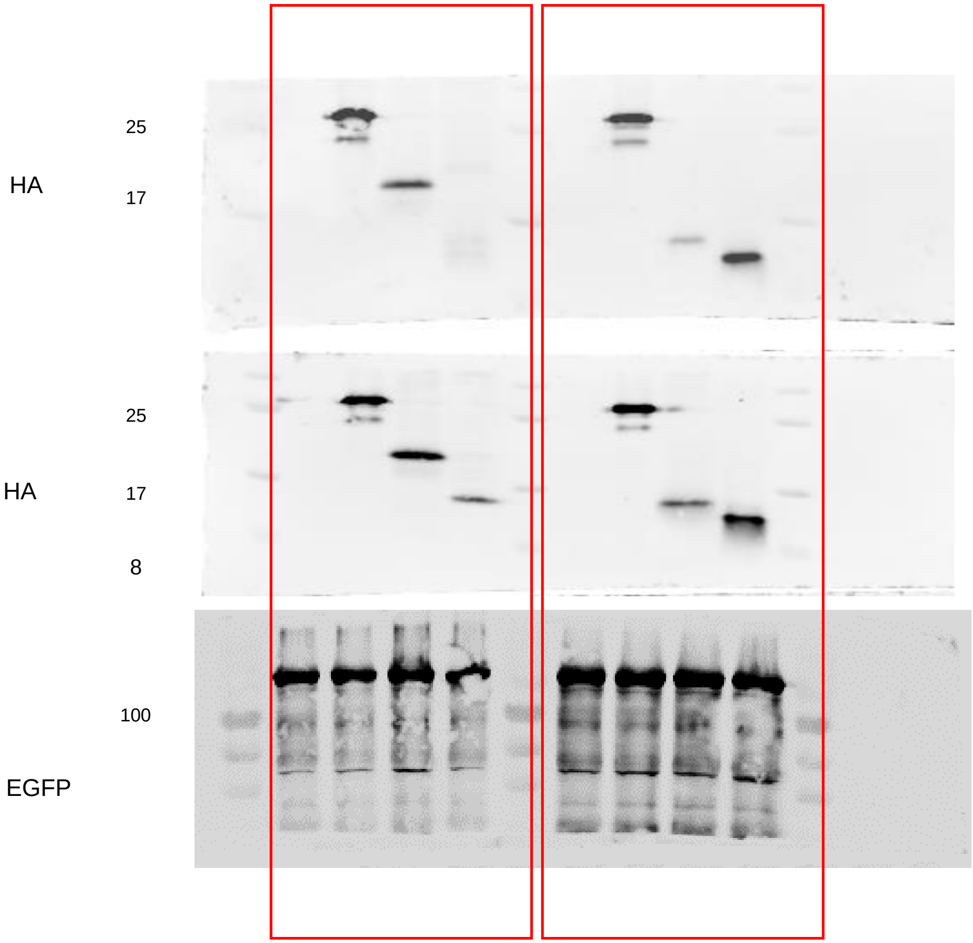

Figure S9H

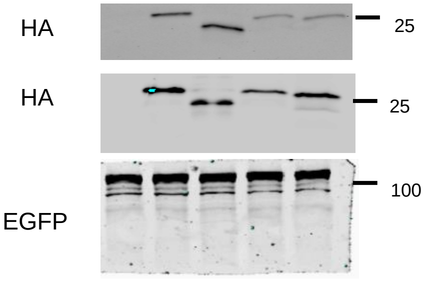

Figure S9G

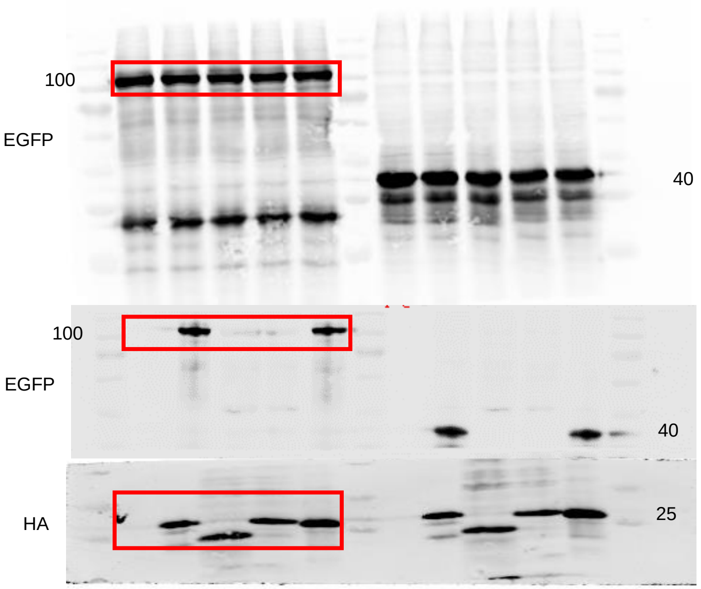

Figure S11D

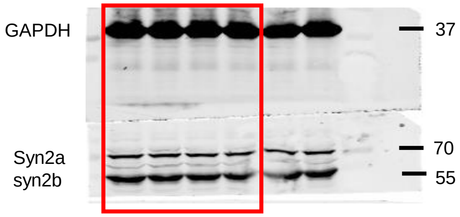

Supplement: Unedited blot and gel images [file jci-134-172802-s100.pdf]
